# Switching metastable dynamics in many-body open quantum systems

**DOI:** 10.1093/nsr/nwag146

**Published:** 2026-03-09

**Authors:** Ya-Xin Xiang, Weibin Li, Zhengyang Bai, Yu-Qiang Ma

**Affiliations:** National Laboratory of Solid State Microstructures and School of Physics, Collaborative Innovation Center of Advanced Microstructures, Nanjing University, Nanjing 210093, China; School of Physics and Astronomy, and Centre for the Mathematics and Theoretical Physics of Quantum Non-equilibrium Systems, University of Nottingham, Nottingham NG7 2RD, UK; National Laboratory of Solid State Microstructures and School of Physics, Collaborative Innovation Center of Advanced Microstructures, Nanjing University, Nanjing 210093, China; National Laboratory of Solid State Microstructures and School of Physics, Collaborative Innovation Center of Advanced Microstructures, Nanjing University, Nanjing 210093, China; Hefei National Laboratory, Hefei 230088, China

**Keywords:** stochastic switching, quantum metastability, dissipative phase transition, Rydberg atom

## Abstract

Stochastic switching is a central phenomenon in dissipative many-body systems, offering a key probe of metastability across classical and quantum regimes. Here, we unravel the connection between switching dynamics and quantum metastability through the lens of spectral decomposition, quantum-jump simulations and the large deviation principles. By establishing a direct correspondence between classical fixed points and quantum metastable states, we distinguish trajectory-level, noise-induced metastability from spectrum-level, deterministic metastability in a Markovian open quantum system with bistability. The Liouvillian gap, the steady-state occupation ratio and the observed switching rates of the metastable states all exhibit exponential scaling with system size, giving rise to a quantum analogue of the Arrhenius law, with the inverse system size serving as an effective temperature. These results provide a unified picture of quantum bistability and clarify the relaxation processes of strongly interacting, dissipative quantum systems far from the thermodynamic limit.

## Teaser

Stochastic switching between metastable states in dissipative many-body systems exhibits a quantum analogue of the Arrhenius law, with the inverse system size acting as an effective temperature.

## INTRODUCTION

Spontaneous switching between metastable states in driven-dissipative quantum many-body systems [1–3] is recently receiving growing attention in quantum science as a key signature of nonequilibrium dissipative phase transitions [4–9], with potential applications in quantum information [10,11] and metrology [12,13]. At first glance, this phenomenon presents an apparent contradiction in Markovian open quantum systems. Governed by the Lindblad equation with a unique stationary state [14], these systems can exhibit a small Liouvillian gap—a hallmark of spectral metastability—near first-order dissipative phase transitions [15–18]. Yet, in finite systems, metastable modes in Liouville space inevitably decay, seemingly at odds with the persistent switching observed in experiments and simulations [3–7].

This apparent contradiction not only highlights the fundamental distinction between the trajectory-level metastability (stochastic switching) and spectrum-level metastability (a small Liouvillian gap), but also raises a crucial question regarding the relation between semiclassical descriptions of phase transitions and quantum metastability in finite systems. In particular, how do the stable fixed points of a mean-field (MF) theory—the stationary solutions to the semiclassical equations of motion—manifest in the full quantum setting?

In finite systems, quantum fluctuations transform these MF fixed points into metastable quantum states with finite lifetimes. These states are mixed through stochastic switching, analogous to how large deviations (LDs) connect coexisting classical basins of attraction [19–21]. Thus, the central open challenge is to establish a quantitative connection between the two facets of quantum metastability: how do the MF fixed points map to the long-lived states spanning the metastable manifold (MM) of Liouville space [22,23], and how does the single spectral gap govern the switching rates between them?

In classical bistable systems, the preference for one metastable state over the other and the exponential scaling of lifetimes with the height of free-energy (or its nonequilibrium counterpart, the quasipotential) barriers—the Arrhenius law—are well understood [19,24]. It is natural to wonder whether this rule applies to quantum bistable systems at zero temperature, and how it emerges from the interplay of spectral properties and stochastic dynamics. In this work, we resolve this question by synthesizing spectral analysis, quantum-jump simulations and semiclassical field theory into a unified framework. Applying this synthesis to a paradigmatic quantum bistable system [18,25]—a Rydberg atom setting [26]—we establish how the exponential scaling of spectral properties directly governs the statistics of stochastic switching.

**Figure 1.**
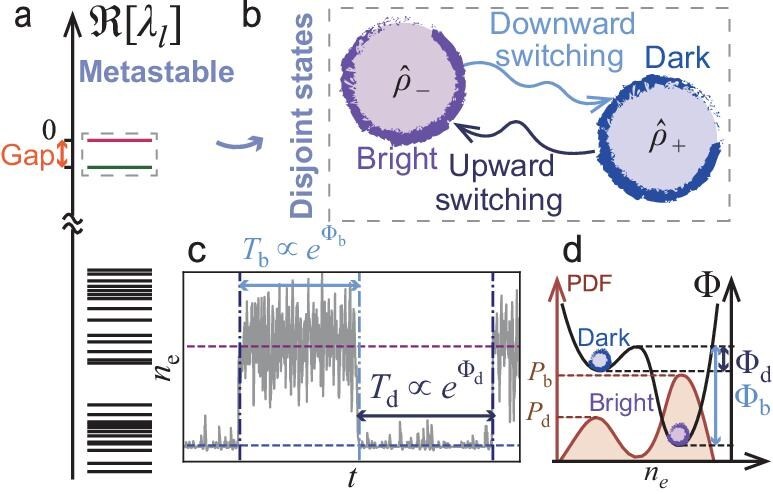
Sketch of quantum metastability and stochastic switching. (a) Quantum metastability arises from both a small spectral gap between a low-lying eigenmode (green line) and the unique stationary state (red line), and a separation between these and the remainder (black lines) in the real part of the Liouvillian spectrum. The first two low-lying modes belong to the metastable manifold (MM), which is spanned by (b) the quantum dark ($\hat{\rho }_+$) and bright ($\hat{\rho }_-$) states, corresponding to the two stable MF fixed points with low and high Rydberg densities, respectively. The dark and bright states are mixed through (c) upward and downward switching with rates $T_{\rm d}^{-1},T_{\rm b}^{-1}$ that follow an Arrhenius-type scaling $T_{\rm b,d}^{-1}\propto e^{-\Phi _{\rm b,d}}$, leading to (d) the stationary probability density function (PDF) $P_{\rm b,d}\propto e^{-\Phi _{\rm b,d}}$, where $\Phi _{\rm b},\Phi _{\rm d}$ are the effective energy barriers quantifying the relative stability of the bright (${\rm b}$) and dark (${\rm d}$) states. The purple and blue horizontal lines indicate the Rydberg population $n_e$ of the two stable MF fixed points, while dark- and light-blue vertical lines in (c) mark the upward and downward switching, respectively.

For this system, MF analysis predicts a first-order dissipative phase transition between states with high and low Rydberg populations, referred to as the bright and dark states, respectively [27]. In finite systems far from the thermodynamic limit, first-order transitions manifest as metastability, with the system exhibiting a small Liouvillian gap (see Fig. 1a). This allows us to extract the two disjoint metastable states that span the MM through spectral decomposition, leveraging the trace-preserving symmetry of the Lindbladian. The resulting states $\hat{\rho }_{-}$ and $\hat{\rho }_+$ in Liouville space directly correspond to their MF counterparts and are identified as the quantum bright and dark states (see Fig. 1b).

Driven by quantum fluctuations, the system switches back and forth between the dark and bright states on timescales significantly exceeding those of microscopic dynamics (see Fig. 1c). Both the switching times (measured from quantum-jump simulations) and the spectral properties—the Liouvillian gap and the steady-state occupation ratio—exhibit exponential scaling with system size, reflecting a quantum analogue of the Arrhenius law.

Viewed through the lens of LDs, the scaling exponents encode the effective energy barriers (Fig. 1d), linking the probabilities of large fluctuations to the steady-state occupation probabilities of the two metastable states [20]. The strong dissipation permits a semiclassical approximation to the long-time dynamics [28]. In the absence of a free-energy functional for nonequilibrium states, we resort to the dynamical path integral [29,30] combined with the instanton approach [20] to determine the effective energy barriers. This allows us to identify the inverse system size as the nonequilibrium analogue of temperature, which also serves as the LD parameter governing the likelihood of such rare fluctuations. Consequently, switching is suppressed in larger systems, consistent with earlier observations [18,31].

The connection between the steady-state occupations and the lifetimes of quantum metastable states provides a key diagnostic for distinguishing metastability from bistability. In a far-off-resonant regime, the system exhibits metastability without bistability. It features a small Liouvillian gap and metastable states, along with large switching-like fluctuations, yet lacks true bistability. The absence of exponential scaling of the steady-state occupation ratio with system size indicates the absence of an Arrhenius law, which is a hallmark of the bistable regime. By extending the classical LD statistics to the quantum realm, our findings offer new insights into quantum bistability while systematically revealing the relaxation dynamics in strongly interacting, dissipative quantum systems.

## MODEL

We consider an ensemble of *N* atoms consisting of two electronic states. Each atom (indexed by the subscript *l*) is continuously laser-excited from the ground state $\mathinner {|{g}\rangle }_l$ to the electronically excited Rydberg state $\mathinner {|{e}\rangle }_l$ with Rabi frequency $\Omega$ and detuning $\Delta$ from resonance. Once excited, the Rydberg states interact via strong, long-range interactions. The microscopic dynamics of the system is described by the Lindblad equation ($\hbar =1$)


(1) \begin{eqnarray*}
\hat{\mathcal {L}}[\hat{\rho }]\equiv \partial _t\hat{\rho }=-i[\hat{H},\hat{\rho }] +\hat{\boldsymbol{D}}[\hat{\rho }].
\end{eqnarray*}

Within the rotating-wave approximation, the resultant Hamiltonian $\hat{H}$ in the interaction picture reads


(2) \begin{eqnarray*}
\hat{H} = \sum _l{\bigg [-\Delta \hat{n}_l+\Omega \hat{\sigma }^{x}_l+\frac{V}{N-1}\sum _{l < k}\hat{n}_k\hat{n}_l\bigg ]},
\end{eqnarray*}

where $\hat{\sigma }_l^{\alpha } (\alpha =x, y, z)$ denote the Pauli matrices acting on the *l*-th atom, and $\hat{n}_{l} =\hat{\sigma }_l^{z}+\mathbf {I}_l/2$ represents the Rydberg number operator. The last term of the Hamiltonian in Equation (2) represents the Rydberg interactions between the *l*-th and *k*-th atoms.

Depending on the selected Rydberg states, excited atoms separated by distance *R* can participate in dipole–dipole (${\sim } R^{-3}$) and van der Waals (vdW) (${\sim } R^{-6}$) interactions [26]. To investigate the long-time dynamics of large systems, we approximate the interaction *V* as a constant all-to-all coupling, normalized to render the Hamiltonian extensive. This approximation is justified in high-density, Rydberg-dressed cold-atom systems [32,33]. Our study is also applicable to other platforms with demonstrated all-to-all interactions, including superconducting quantum simulators [34,35] and cavity-assisted atomic systems [36–38].

Each atom couples to the local environment, which is assumed to be in the vacuum state, resulting in a finite lifetime and the dissipative dynamics


(3) \begin{eqnarray*}
\hat{\boldsymbol{D}}[\hat{\rho }]=\frac{\gamma }{2}\sum _{l}{ (2\hat{\sigma }_l^{-}\hat{\rho }\hat{\sigma }_l^{+}- \lbrace \hat{\sigma }_l^{+}\hat{\sigma }_l^{-},\hat{\rho }\rbrace )},
\end{eqnarray*}

where the operators $\hat{\sigma }_l^\pm \equiv \hat{\sigma }_l^{x}\pm i\hat{\sigma }_l^{y}$ flip the atomic state. Equation (3) describes the decay processes from $\mathinner {|{e}\rangle }$ to $\mathinner {|{g}\rangle }$ with rate $\gamma$.

## RESULTS

### Mean-field analysis

We begin with an MF ansatz $\hat{\rho }=\prod _{l}{\otimes \hat{\rho }_l}$ to characterize the dynamical behavior of this system in the thermodynamic limit. Introducing the collective spin operators ($\alpha =x, y, z$) $\hat{S}^\alpha =\sum _l{\hat{\sigma }^\alpha _l}$, we obtain the following set of coupled nonlinear equations for the expectation values $m_\alpha \equiv {\rm Tr}{[\hat{S}^\alpha \hat{\rho }]/S}$ (with the total spin $S=N/2$):


(4a) \begin{eqnarray*}
\partial _t m_x = -\bigg [\frac{V}{2}( m_z+1)-\Delta \bigg ]m_y - \frac{\gamma }{2}m_x ,
\end{eqnarray*}

(4b) \begin{eqnarray*}
\partial _t m_y = -\Omega m_z + \bigg [\frac{V}{2}(m_z+1)-\Delta \bigg ]m_x - \frac{\gamma }{2}m_y,\\
\end{eqnarray*}

(4c) \begin{eqnarray*}
\partial _t m_z = \Omega m_y - \gamma (m_z+1) ,
\end{eqnarray*}

where the Rydberg population $n_e\equiv {\rm Tr}[{\sum _l{\hat{n}_{l}}\hat{\rho }}]/N=(m_{z}+1)/2$. Without loss of generality, we choose $\gamma ^{-1}$ as the time unit and fix Rabi frequency $\Omega =1.5$ and the atomic interaction $V=10$.

**Figure 2.**
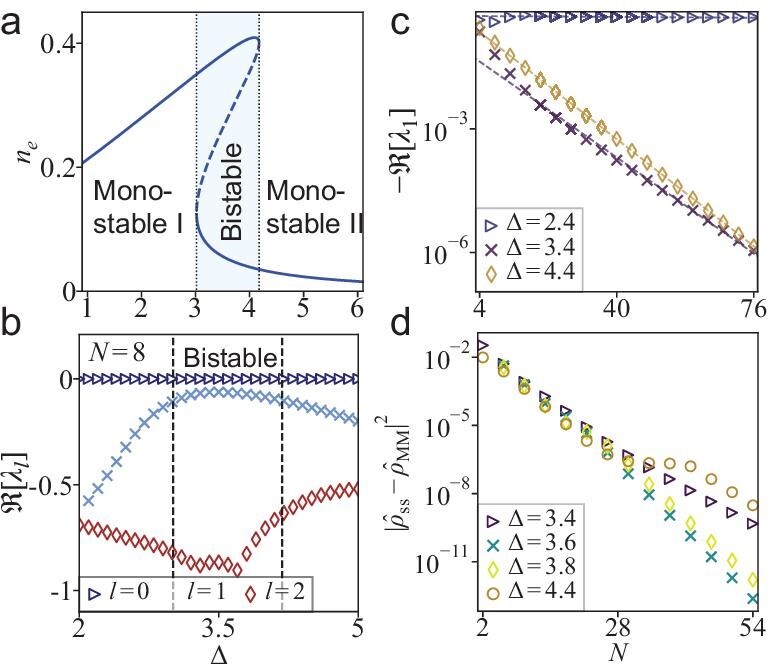
(a) MF stable (unstable) fixed points as a function of detuning $\Delta$, represented by solid (dashed) lines. Phase boundaries are highlighted by dotted gray lines, and the bistable region in between is shaded blue. (b) Real part of the Liouvillian eigenvalues for $N=8$. The index *l* labels the eigenvalues. The Liouvillian eigenvalues $\lambda _l$ are arranged by their real parts in descending order, and $\lambda _0=0$. The bistable regime is located between the two black dashed lines. (c) Finite-size scaling for the spectral gap in the monostable I ($\Delta$=2.4), bistable ($\Delta =3.4$) and monostable II ($\Delta =4.4$) regimes. Dashed lines are obtained from fitting $-\Re [\lambda _1]=b e^{a N}$. (d) Errors of the approximation of the stationary state $\hat{\rho }_{\rm ss}$ by the linear combination of the metastable disjoint subspaces, $\hat{\rho }_{\rm MM}\equiv \sum _{\alpha =\pm }{D[\hat{\rho }_\alpha ,\hat{\rho }_{\rm ss}]\hat{\rho }_\alpha }$, with coefficients given by the normalized Hilbert–Schmidt inner products $D[\hat{A},\hat{B}]\equiv \mathinner {\langle {\hat{A}^\dagger ,\hat{B}}\rangle }/\mathinner {\langle {\hat{A}^\dagger ,\hat{A}}\rangle }$, where $\vert {\hat{A}}\vert ^2\equiv \mathinner {\langle {\hat{A}^\dagger ,\hat{A}}\rangle }$ and $\mathinner {\langle {\hat{A},\hat{B}}\rangle }\equiv {\rm Tr}{[\hat{A} \hat{B}]}$. Results are shown for the bistable ($\Delta =3.4,3.6,3.8$) and monostable II ($\Delta =4.4$) regimes.

Linear stability analysis of the fixed points derived from Equations (4a)–(4c) reveals that the system exhibits a first-order phase transition as a function of detuning. As illustrated in Fig. 2a, based on the number of stable fixed points, the phase diagram is partitioned into three distinct regimes: monostable I, monostable II and bistable. In the bistable regime, each of the two stable fixed points possesses a unique basin of attraction, and the system evolves towards either of the two stable fixed points, determined by the initial conditions.

### Quantum metastable states

Phase transitions strictly occur in the thermodynamic limit; in finite systems, they are instead characterized by the emergence of metastability. Quantum fluctuations destabilize the MF fixed points, replacing them with metastable quantum states of finite lifetimes.

Since the all-to-all coupling preserves the permutation symmetry, we rewrite the Hamiltonian in Equation (2) and the dissipation in Equation (3) in terms of collective Dicke states $\mathinner {|{M}\rangle }$. Thus, we obtain the effective non-Hermitian Hamiltonian


(5) \begin{eqnarray*}
\hat{H}_{\rm eff}=\Omega \hat{S}^x\!-\!\frac{V}{2N}\hat{S}^{+}\hat{S}^{-} +\bigg (\frac{V-i}{2}\!-\!\Delta \bigg )\hat{S}^z-\frac{iS}{2},
\end{eqnarray*}

where the operators $\hat{S}^{\pm }=\sum _{l}{\hat{\sigma }_l^{\pm }}$ satisfy $\hat{S}^{\pm }\mathinner {|{M}\rangle }=\sqrt{(S\mp M)(S\pm M+1)}\mathinner {|{M\pm 1}\rangle }$ and the commutation relation $[\hat{S}^+,\hat{S}^-]=2\hat{S}^z$. We propose the heuristic jump operator $\hat{L}=\sum _{M}{\sqrt{M+S}\mathinner {|{M-1}\rangle }\mathinner {\langle {M}|}}$, which preserves the permutation symmetry of the dissipative dynamics in Equation (3) and reproduces the linear dependence of the collapse probability on the Rydberg population, while neglecting the phase differences induced by local quantum jumps (see Section SI)

We are now in a position to explore signatures of first-order phase transitions in finite systems by examining the low-lying modes of the Liouvillian superoperator $\hat{\mathcal {L}}$. We obtain its eigenvalues and eigenmatrices via $\hat{\mathcal {L}}[\hat{\rho }_l]=\lambda _l\hat{\rho }_l$ and sort them according to $0=\lambda _0\ge \Re [\lambda _1]\ge \cdots \ge \Re [\lambda _{(N+1)^2}]$. It follows from $\lambda _0=0$ that the stationary density matrix is $\hat{\rho }_{\rm ss}=\hat{\rho }_0/ {\rm Tr}{[\hat{\rho }_0]}$, and the Liouvillian spectral gap is defined as $-\Re [\lambda _1]$. The real parts of the first three eigenvalues are displayed in Fig. 2b, where the spectral gap decreases as the system approaches the bistable regime. After plotting the gap in Fig. 2c, we find that the gap is independent of system size for small $\Delta$ (monostable I). However, for large $\Delta$ (bistable and monostable II), the gap decreases exponentially with *N*.

The eigenvalue $\lambda _1$ is complex for small detuning and becomes real as it approaches the bistable regime (see Section SI). For the case where $\lambda _1$ is real, the separation in the lifetimes of the first two eigenmodes $\hat{\rho }_{{\rm ss}}$ and $\hat{\rho }_{1}$ suggests a double degeneracy, whereby the MM is spanned by a pair of orthonormal bases (density matrices), denoted $\hat{\rho }_\pm$ (with ${\rm Tr}{[\hat{\rho }_\pm ]}=1$ and $\hat{\rho }_\pm =\hat{\rho }_\pm ^\dagger$). It follows that $\hat{\rho }_1=a_+\hat{\rho }_{+}+a_{-}\hat{\rho }_{-}$. Because the Lindblad equation preserves the trace (probabilities), any eigenmatrices with nonzero eigenvalues are traceless. Thus, we obtain $a_{+}=-a_{-}$. In addition, it follows from the Lindblad Equation (1) that, for any Liouvillian, if $\hat{L}[\hat{\rho }] = \lambda \hat{\rho }$ then $\hat{L}[\hat{\rho }^\dagger ] = \lambda ^\ast \hat{\rho }^\dagger$. Therefore, when $\lambda _1$ is real and of degeneracy 1, the eigenmatrix $\hat{\rho }_1$ is Hermitian (i.e. $a_{\pm }\in \mathbb {R}$) and can be diagonalized, yielding the spectral decomposition [15]


(6) \begin{eqnarray*}
\hat{\rho }_1 &=&\sum _{l}{\theta (\alpha _l)\alpha _l\mathinner {|{\alpha _l}\rangle }\mathinner {\langle {\alpha _l}|}}\\
&& -\sum _{l}{\theta (-\alpha _l)(-\alpha _l)\mathinner {|{\alpha _l}\rangle }\mathinner {\langle {\alpha _l}|}},
\end{eqnarray*}

where $\theta (x)$ is the Heaviside step function, and all eigenvalues $\alpha _l$ are real, with $\left\langle \alpha _l \middle | \alpha _k \right\rangle = \delta _{l,k}$.

One immediately recognizes the two density matrices $\hat{\rho }_{\pm }$ as the subspaces spanned by the eigenvectors (pure states) of $\hat{\rho }_1$ with positive ($+$) and negative ($-$) eigenvalues, i.e.


(7a) \begin{eqnarray*}
\hat{\rho }_- \propto \sum _{l}{\theta (-\alpha _l)(-\alpha _l)\mathinner {|{\alpha _l}\rangle }\mathinner {\langle {\alpha _l}|}},
\end{eqnarray*}

(7b) \begin{eqnarray*}
\hat{\rho }_+ \propto \sum _{l}{\theta (\alpha _l)\alpha _l\mathinner {|{\alpha _l}\rangle }\mathinner {\langle {\alpha _l}|}},
\end{eqnarray*}

followed by normalization ensuring that ${\rm Tr}{[\hat{\rho }_\pm ]}=1$. We note that the two subspaces are, by construction, orthogonal, i.e. $\mathinner {\langle {\hat{\rho }_+,\hat{\rho }_-}\rangle } =0$, where the Hilbert–Schmidt inner product (for operators $\hat{A}$ and $\hat{B}$) is defined as $\mathinner {\langle {\hat{A},\hat{B}}\rangle }\equiv {\rm Tr}{[\hat{A}\hat{B}]}$. They therefore constitute the disjoint subspaces of the MM associated with the slowest relaxation [15,23,39]. We project the stationary state $\hat{\rho }_{ss}$ onto the MM through $\mathcal {P}_{\rm MM}[\hat{\rho }_{\rm ss}]\!\equiv\! \hat{\rho }_{\rm MM}=\sum _{\alpha =\pm }{D[\hat{\rho }_\alpha ,\hat{\rho }_{\rm ss}] \hat{\rho }_\alpha }$, where the normalized inner product is $D[\hat{A},\hat{B}]\!\equiv\! \mathinner {\langle {\hat{A}^\dagger , \hat{B}}\rangle }/\mathinner {\langle {\hat{A}^\dagger , \hat{A}}\rangle }$. As sketched in Fig. 2d, in both the bistable and monostable II regimes, the error $\vert {\hat{\rho }_{\rm ss}-\hat{\rho }_{\rm MM}}\vert ^2$ is small and also decreases exponentially with *N*.

**Figure 3.**
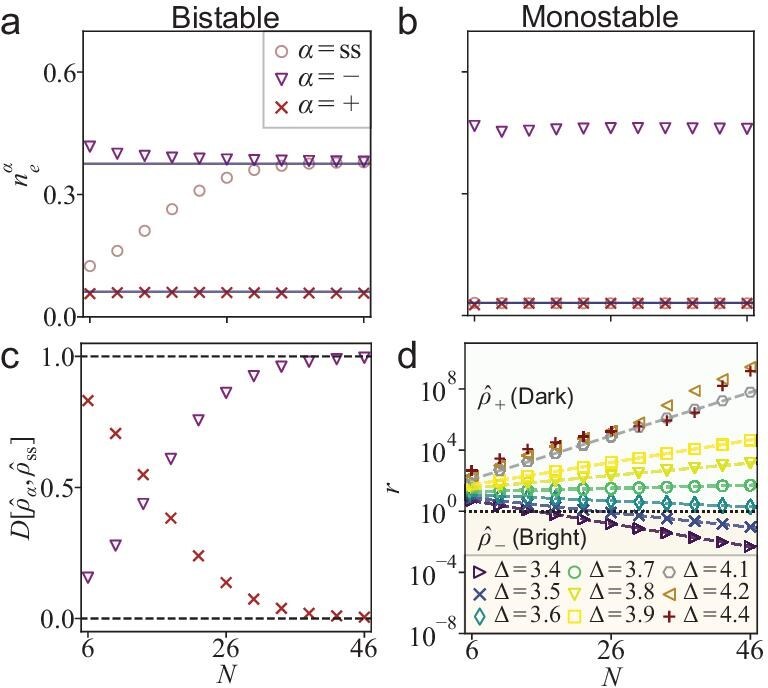
Average excited population according to the density matrices $\hat{\rho }_{{\rm ss}}$ and $\hat{\rho }_{\pm }$ as a function of *N* for (a) $\Delta =3.4$ (bistable) and (b) $\Delta =4.4$ (monostable II). The blue lines represent the MF stable fixed points. (c) Normalized Hilbert–Schmidt inner products $D[\hat{\rho }_{\pm },\hat{\rho }_{{\rm ss}}]$ between $\hat{\rho }_{\pm }$ and the steady state $\hat{\rho }_{{\rm ss}}$ as a function of *N* for $\Delta =3.4$. The black dashed lines represent the upper and lower bounds. (d) Ratio $r = D[\hat{\rho }_{+},\hat{\rho }_{\rm ss}]/D[\hat{\rho }_{-},\hat{\rho }_{\rm ss}]$ of the steady-state occupation probabilities of the dark and bright states for the bistable ($\Delta = 3.4$–4.1) and monostable II ($\Delta =4.2, 4.4$) regimes. Dashed lines show nonlinear fits of the form $c e^{a N}$.

The relation between the two metastable subspaces $\hat{\rho }_\pm$ and the two stable MF fixed points is elucidated by examining the average excitation densities ($\alpha ={\rm ss},\pm$) $n_e^{\alpha }={\rm Tr}{[\hat{n}_e\hat{\rho }_{\alpha }]}$. Within the bistable regime ($\Delta =3.4$; Fig. 3a and c), the two metastable states $\hat{\rho }_\pm$ show excitation densities identical to those of the MF fixed point, along with distinct photon-emission statistics (see Section SII). The steady-state excitation population $n_e^{\rm ss}$ is a statistical mixture of the dark and bright states. In the thermodynamic limit ($N\rightarrow \infty$), it asymptotically approaches the bright (dark) state for small (large) detuning (see Section SII). This asymptotic behavior produces a discontinuous jump in $n_e^{\rm ss}$ at a critical detuning $\Delta _{\rm c}$—a hallmark of first-order phase transition. Therefore, we designate the states $\hat{\rho }_{+}$ and $\hat{\rho }_{-}$ as *the quantum dark and bright states*, respectively.

### Bistability versus metastability

To quantify the relative probability of finding the system in one metastable state relative to the other in the bistable regime. We define the ratio $r=D[\hat{\rho }_+,\hat{\rho }_{\rm ss}]/D[\hat{\rho }_{-},\hat{\rho }_{\rm ss}]$ to characterize the relative probability, where $r\gg 1$ ($r\ll 1$) indicates that the system is predominantly in the dark (bright) state. As shown in Fig. 3d, the ratio *r* provides a key diagnostic for distinguishing the bistable and monostable II regimes. Both regimes have a real $\lambda _1$ and host metastable states $\hat{\rho }_\pm$ (Fig. 3a and b), yet their occupation statistics differ significantly. In the bistable regime, *r* varies exponentially with the system size *N* (with a detuning-dependent exponent). In the monostable II regime, this exponential scaling breaks down. This distinction underscores the difference between metastability at the spectral level (governed by the Liouvillian gap) and at the trajectory level (which also requires exponentially scaling occupations).

The exponential size scaling of *r* implies a corresponding exponential scaling of the switching times. We extract these switching times by keeping track of the upward and downward switching events from the simulated trajectories (see Section SIII for exemplary trajectories). In the simulation, given the time step $dt$, at each step the wave function $\mathinner {|{\psi _t}\rangle }$ either collapses, $\mathinner {|{\psi _t}\rangle }\rightarrow {\hat{L}\mathinner {|{\psi _t}\rangle }}/{\sqrt{\mathinner {\langle {\psi _t}|}\hat{L}^{\dagger }\hat{L}\mathinner {|{\psi _t}\rangle }}}$, with probability $P_t=dt\mathinner {\langle {\psi _t}|}\hat{L}^{\dagger }\hat{L}\mathinner {|{\psi _t}\rangle }$, or evolves under the action of the effective non-Hermitian Hamiltonian, $\mathinner {|{\psi _t}\rangle }\rightarrow {e^{-i\hat{H}_{\rm eff}dt}\mathinner {|{\psi _t}\rangle }}/{\sqrt{1-P_t}}$, with probability $1-P_t$. The time-dependent average Rydberg population is computed according to $n_e(t)=\mathinner {\langle {\psi _t}|}\hat{S}^z\mathinner {|{\psi _t}\rangle }/N+0.5$ [27,40,41].

**Figure 4.**
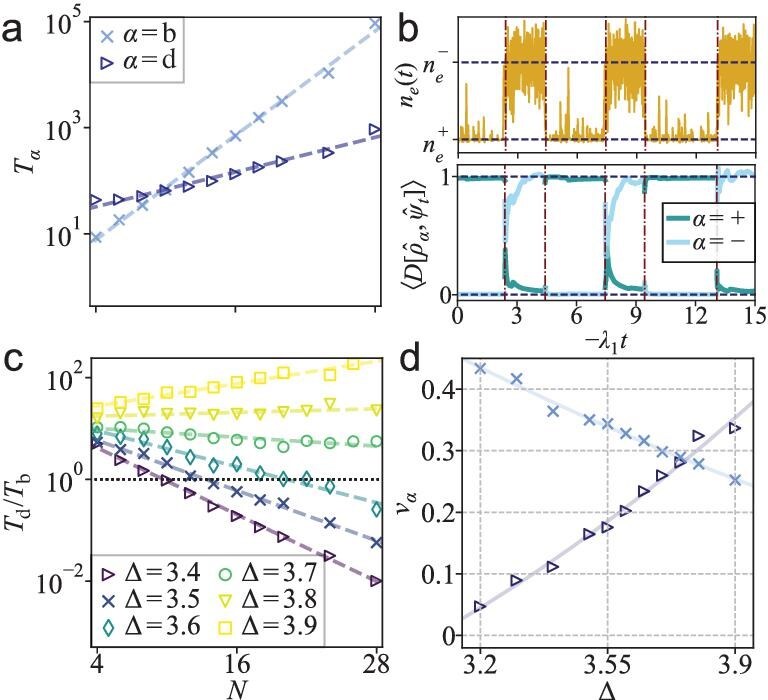
(a) Mean waiting times for the dark ($T_{\rm d}$) and bright ($T_{\rm d}$) states as a function of the number of atoms *N* for $\Delta = 3.4$. (b) Quantum trajectory for $\Delta =3.4,N=10$, where red dashed lines mark reset events at each quantum jump. Upper: average excitation population of the trajectory $\mathinner {|{\psi _t}\rangle }$. Blue dashed lines indicate the excitation densities $n_e^\pm$ of the two quantum metastable states $\hat{\rho }_\pm$. Lower: time-averaged normalized Hilbert–Schmidt inner products of the instantaneous pure state $\hat{\psi }_t = \mathinner {|{\psi _t}\rangle } \mathinner {\langle {\psi _t}|}$ with respect to $\hat{\rho }_+$ (blue) and $\hat{\rho }_-$ (green), computed between successive switching events. (c) Ratio of the mean waiting times for upward to downward switching for varying detuning and system size. (d) Effective energy barrier $v_{\rm db}$ for the dark and bright states extracted from fitting the waiting times with $T_\alpha = c_\alpha e^{v_\alpha N}$ ($\alpha = {\rm b,d}$), where the exponent $v_\alpha$ corresponds to the slope of the curves in panel (a).

A comparison of the mean switching times $T_{\rm d}$ and $T_{\rm b}$ across different numbers of atoms *N* with fixed detuning $\Delta = 3.4$ is given in Fig. 4a. Both $T_{\rm d}$ and $T_{\rm b}$ increase exponentially with *N*, albeit with different exponents. Consequently, although the dark state is preferred for small *N*, the waiting time (lifetime) of the bright state increases with *N* more rapidly than that of the dark state, indicating that the bright state becomes more stable in larger systems.

To further elucidate the relationship between trajectory- and spectrum-level metastability, we consider a quantum trajectory with reset. After each stochastic switching event, the density operator $\hat{\rho }_t$ is reset to that of the instantaneous pure state $\hat{\psi }_t = \mathinner {|{\psi _t}\rangle }\mathinner {\langle {\psi _t}|}$. In the time interval between successive switching events, the ensemble density operator evolves as the average over all such pure states following the last reset.

As shown in Fig. 4b, following each stochastic switching, there is a coordinated sharp change in both the excitation density $n_e(t)$ and the occupation $D[\hat{\rho }_\pm , \hat{\psi }_t]$. The dynamics of the occupation between successive resets reveals a fast relaxation (on timescales $\lesssim -\Re [\lambda _2]^{-1}$), followed by a latent period (on timescales ${\sim } -\lambda _1^{-1}$). During this latent period, the time-averaged density operator follows $\hat{\rho }(t) = \hat{\rho }_{\rm ss} + A(\hat{\rho }_+ - \hat{\rho }_-)e^{\lambda _1 t}$. Therefore, after each upward (downward) switching event, the coefficient *A* is reset to $-D[\hat{\rho }_+,\hat{\rho }_{\rm ss}](D[\hat{\rho }_-,\hat{\rho }_{\rm ss}]$).

This shows that spectral-level metastability manifests whenever switching occurs, and the stationary state $\hat{\rho }_{\rm ss}$ emerges dynamically from stochastic switching between the metastable states $\hat{\rho }_\pm$. Accordingly, the switching rates $T_{\rm b}^{-1}$ and $T_{\rm d}^{-1}$ are proportional to $-\lambda _1 D[\hat{\rho }_+,\hat{\rho }_{\rm ss}]$ and $-\lambda _1 D[\hat{\rho }_-,\hat{\rho }_{\rm ss}]$, respectively (see Section SVI for a quantitative comparison with simulations). The combined effects of metastability and bistability bring about dynamic hysteresis in large systems [16,18,31,42,43].

As evinced in Fig. 4c, parallel to the occupation probabilities (see Fig. 4a), the ratio of the two switching rates $T_{\rm d}/T_{\rm b}$ also varies exponentially with the system size, with deviations associated with the uncertainties in determining the bright and dark states from fluctuating data. The relation between the steady-state occupation probabilities and the waiting times of the two quantum states reflects the essence of their dynamical coexistence, providing the physical basis for the emergence of a unique steady state from two metastable states. Through log-linear fitting of the waiting times $T_\alpha \propto e^{v_\alpha N}$ ($\alpha = {\rm b,d}$), we can estimate the effective energy barrier $v_\alpha$ for each state. It is seen from Fig. 4d that the energy barrier for the dark (bright) state increases (decreases) with the detuning.

### Instantons and the Arrhenius-like scaling

We now explicate how the exponential size scaling of the relative occupation probabilities as well as the mean switching times reflects an Arrhenius-type exponential scaling with the effective energy barrier for escaping from the two metastable states.

For Lindbladian dynamics, the quantum noise stems from the corresponding quantum Heisenberg–Langevin equations [40], where, on top of the deterministic dynamics generated by the effective non-Hermitian Hamiltonian, there are additional stochastic dynamics induced by the quantum noise operators. The strong dissipation of the system allows for a semiclassical approximation to its long-time switching dynamics. In this regime, the Heisenberg–Langevin equations for operators reduce to classical Langevin equations for their expectation values [28] (see Section SIV). The resultant Langevin equations can be cast into a dynamic path integral via a Martin–Siggia–Rose construction [29,30] with the partition function (see Section SV)


(8) \begin{eqnarray*}
\mathcal {Z}=\int {\mathcal {D}[m_x,m_y,m_z,\tilde{m}_z,\tilde{m}_y, \tilde{m}_z] e^{-\mathcal {S}}},
\end{eqnarray*}

with the nonequilibrium action (the sum over repeated indices is implied hereinafter)


(9) \begin{eqnarray*}
\mathcal {S} =\int {dt \bigg \lbrace \tilde{m}_\alpha \bigg (\partial _t m_\alpha - F_\alpha - \frac{M_{\alpha \beta }}{2}\tilde{m}_\beta \bigg )\bigg \rbrace },
\end{eqnarray*}

where $\alpha ,\beta \in \lbrace x,y,z\rbrace$, the generalized force $F_\alpha$ is given by Equation (4), and the covariance $M_{\alpha \beta }$ for the white, zero-mean Langevin noise $\eta _{\alpha ,t}$ is fixed via the generalized Einstein relation [28,44], i.e.


\begin{eqnarray*}
\mathinner {\langle {\eta _{\alpha ,t}\eta _{\beta ,t^{\prime }}}\rangle }=\frac{\delta (t-t^{\prime })}{2N} \mathinner {\langle {\partial _t (\hat{S}^\alpha _t\hat{S}^\beta _t) -\partial _t(\hat{S}^\alpha _t)\hat{S}^\beta _t -\hat{S}^\alpha _t\partial _t(\hat{S}^\beta _t) + {\rm H.c.}}\rangle },
\end{eqnarray*}

with H.c. representing the Hermitian conjugate. By introducing the vectorial notation $\boldsymbol{\eta }_t=(\eta _{x,t},\eta _{y,t},\eta _{z,t})^T$, the covariance matrix $\boldsymbol{M}_t\delta (t-t^{\prime })=\mathinner {\langle {\boldsymbol{\eta }_t\boldsymbol{\eta }^T_{t^{\prime }}}\rangle }$ is explicitly written as (time dependence made implicit)


(10) \begin{eqnarray*}
\boldsymbol{M}=\frac{1}{N}
\left({\begin{array}{ccc}
1&\quad 0&\quad m_x\\
0&\quad 1&\quad m_y\\
m_x&\quad m_y &\quad 2(m_z+1) \end{array}}\right).
\end{eqnarray*}

By including the response fields $\tilde{m}_\alpha$, the phase-space dimension increases from 3 to 6. We now introduce the momentum $q_\alpha =\tilde{m}_\alpha /N$ conjugate to $m_\alpha$ and assign a vector $\boldsymbol{x}=(m_x,m_y,m_z,q_x,q_y,q_z)^T$ to each state in phase space.

The optimal transition trajectory is obtained through the saddle-path approximation to the action in Equation (9) with starting and ending points fixed [21,45,46]. This leads to the Hamilton–Jacobi equations for the coordinates $m_\alpha$ and momenta $q_\alpha$:


(11a) \begin{eqnarray*}
\partial _t m_\alpha = \partial _{q_\alpha }H_{\rm cl},
\end{eqnarray*}

(11b) \begin{eqnarray*}
&\partial _t q_\alpha = - \partial _{m_\alpha }H_{\rm cl}.
\end{eqnarray*}

Here the classical Hamiltonian $H_{\rm cl}$ is given by


(12) \begin{eqnarray*}
H_{\rm cl}=F_\alpha q_\alpha + \frac{1}{2}\bar{M}_{\alpha \beta } q_\alpha q_\beta
\end{eqnarray*}

with the rescaled noise covariance $\bar{M}_{\alpha \beta }=NM_{\alpha \beta }$.

After inserting Equations (11) and (12) into Equation (9), the action associated with the transition from state $\boldsymbol{x}_a$ at time $t=0$ to state $\boldsymbol{x}_b$ after a finite time $T\ge 0$ reads


(13) \begin{eqnarray*}
\mathcal {S}_{T} &=& N \int _0^T{dt ( q_\nu \partial _t m_\nu - H_{\rm cl} )}\\
&=&\frac{N}{2}\int _0^T{dt ( q_\nu M_{\nu \alpha }q_\alpha )},
\end{eqnarray*}

which vanishes for deterministic dynamics ($q_\alpha =0$) and is positive when fluctuations are required ($q_\alpha \ne 0$), as is the case for switching between metastable states. Because Hamiltonian (12) is an integral of motion, the action (13) can be parameterized with ‘energy’ *E*:


(14) \begin{eqnarray*}
\mathcal {S}_{T,E} =N \bigg ( \int _{a}^{b}{q_\nu d m_\nu }-ET\bigg ).
\end{eqnarray*}

We also obtain the effective energy barrier (quasipotential) $\Phi (\boldsymbol{x}_a\rightarrow \boldsymbol{x}_b)$ from state $\boldsymbol{x}_a$ to $\boldsymbol{x}_b$, which is defined as the accumulated action along the optimal paths (the so-called instantons) [19–21,45,46]:


(15) \begin{eqnarray*}
\Phi (\boldsymbol{x}_a\rightarrow \boldsymbol{x}_b) \equiv \inf _{T>0}{\inf {\mathcal {S}_{T,E}}},
\end{eqnarray*}

where the minimization is taken over all paths ${\boldsymbol{x}(t)}$ with the boundary conditions $\boldsymbol{x}(0)=\boldsymbol{x}_a$ and $\boldsymbol{x}(T)=\boldsymbol{x}_b$ ($t\in [0,T]$). It follows from Equation (8) that the asymptotic transition rate $\Gamma _{\boldsymbol{x}_a\rightarrow \boldsymbol{x}_b}$ from state $\boldsymbol{x}_a$ to $\boldsymbol{x}_b$ takes the LD form


(16) \begin{eqnarray*}
\Gamma _{\boldsymbol{x}_a\rightarrow \boldsymbol{x}_b} \approx e^{-\Phi (\boldsymbol{x}_a\rightarrow \boldsymbol{x}_b)}.
\end{eqnarray*}

As indicated by Equation (14), the action scales linearly with the system size, thereby indicating an exponential scaling as discussed above. As a result, the rate $\Gamma _{\boldsymbol{x}_a\rightarrow \boldsymbol{x}_b}$ asymptotically approaches 0 (1) as *N* approaches infinity (zero). Additionally, the quasipotential $\Phi$ is time independent, implying that the instantons are also time invariant. Since the time *T* in Equation (14) is not fixed, the instanton can be rendered stationary only when $E=0$ [45,46]. The three fixed points $\boldsymbol{x}_b$, $\boldsymbol{x}_d$ and $\boldsymbol{x}_u$, which nullify Equation (11), are located on the zero-energy manifold and correspond to the MF bright, dark and unstable states, respectively. Consequently, a trajectory that connects any two of them and evolves with time according to Equations (11) is the instanton.

**Figure 5.**
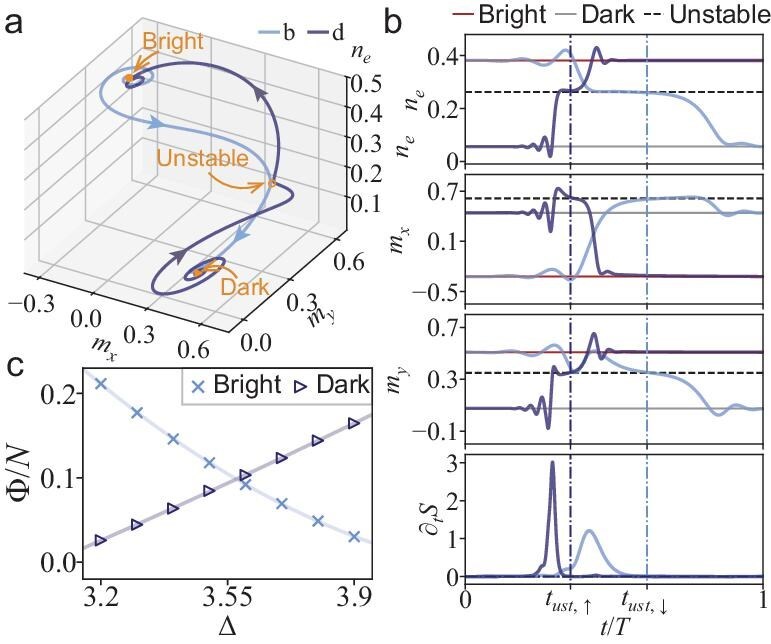
Optimal switching paths (instantons) obtained within the Martin–Siggia–Rose dynamical path-integral formalism after the semiclassical approximation. (a) The instantons for switching from the dark (bright) state to the bright (dark) state for $\Delta =3.5$. The dot, triangle and circle represent the bright, dark and unstable states, respectively. The arrows along the curves indicate the direction of time. (b) The spin fields $n_e, m_x$ and $m_y$, and the action-increment rate along the instantons in (a) as a function of the rescaled time $t/T$, where *T* is the total evolution time. The dashed (solid) horizontal lines denote the unstable (stable) MF fixed point(s), while the vertical lines indicate the times at which the system reaches the unstable state. (c) The effective energy barrier (quasipotential) $\Phi$, defined as the increment of the action along the instanton divided by the system size *N*, as a function of detuning $\Delta$ for the dark and bright states.

We utilize the minimum-action method [20] to find the instanton for switching (see Section SV). The resulting trajectories of upward and downward switching, which correspond to the instantons for escaping from the dark and bright states, are displayed in Fig. 5a. The time series of the spin fields and the action-increment rate $\partial _t\mathcal {S}$ associated with these paths are shown in Fig. 5b. As indicated by Equation (13), escaping from the respective initial attractive basins requires nonzero fluctuations, thereby resulting in action increments for both upward and downward switching. Upon reaching the unstable fixed point (highlighted with vertical dashed lines), the system relaxes towards another attractive basin along deterministic paths, which are devoid of fluctuations and action increments [19].

The effective energy barrier normalized by the system size, calculated according to Equations (14) and (15), is plotted in Fig. 5c. It is evident that the energy barrier for the bright (dark) state decreases (increases) with increasing detuning $\Delta$. This trend results in an increasing (decreasing) escape rate for the bright (dark) state. Such behavior is consistent with the increase in the ratio of the steady-state occupation probability and the switching time of the dark state to that of the bright state (see Fig. 4a and c).

### Switching under van der Waals interaction

**Figure 6.**
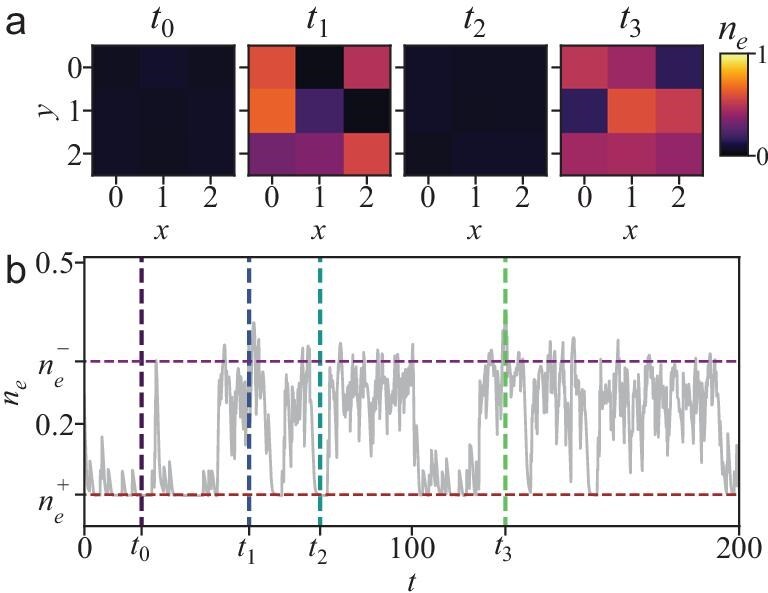
Trajectory dynamics of a $3\times 3$ system with van der Waals interactions for $\Delta =3.4$. (a) Site-resolved and (b) spatially averaged excitation density $n_e$. Vertical dashed lines indicate the times of the snapshots shown in (a). The excitation density $n_e^\pm$ of the two quantum metastable states $\hat{\rho }_\pm$ are marked by red and purple dashed lines.

To enable systematic finite-size scaling, we employ all-to-all interactions, which reduce the computational complexity from $\mathcal {O}(4^N)$ to $\mathcal {O}(N^4)$ while retaining experimental relevance in high-density setups. The connection between spectral- and trajectory-level metastability extends beyond this simplified limit. We have preformed additional calculations for systems with vdW interactions, confirming that the switching dynamics persists. As shown in Fig. 6, the Liouvillian spectrum in such system exhibits a real eigenvalue $\lambda _1$, whose corresponding eigenmatrix satisfies $\hat{\rho }_1\propto \hat{\rho }_+-\hat{\rho }_-$. The excitation densities $n_e^\pm$ of the states $\hat{\rho }_\pm$ remain localized near the MF dark and bright states and are insensitive to the system size (see Section SVII). Individual trajectories display clear stochastic switching between these dark and bright states, accompanied by correlated excitation and de-excitation of atoms.

## CONCLUSION

In this work, we systematically investigate the connection between quantum bistability and stochastic switching in a system exhibiting a first-order dissipative phase transition in the thermodynamic limit. We demonstrate that, in finite systems, discontinuous phase transitions emerge through quantum metastability, with distinct features in both the statistics of quantum fluctuations and the low-lying eigenmodes of the Liouvillian. Exploiting spectral decomposition, we establish a direct correspondence between classical fixed points and quantum metastable states.

We find that quantum bistability has its origin not only in the closing of the spectral gap but also in the exponential size scaling of the steady-state partition of two disjoint quantum metastable states. The former is a signature of quantum metastability at the spectrum level, whereas the latter indicates quantum metastability at the trajectory level, which is characterized by an Arrhenius-type scaling of stochastic switching between these states.

Our results generalize the Arrhenius scaling of the decay rates of homogeneous metastable states to quantum systems, establishing a connection between classical stochastic dynamics and quantum bistability. Crucially, their purely quantum origin distinguishes the switching from its classical counterparts. Occurring at zero absolute temperature, the stochastic switching is purely driven by quantum fluctuations. Like their equilibrium counterparts, the two metastable states differ in their robustness to fluctuations, which is manifested in their steady-state occupation probabilities and switching rates. Through a semiclassical instanton approach and quantum-jump simulations, we reveal that the mean times between successive switching events diverge exponentially with system size, with different exponents reflecting the effective potential barriers for each state. Moreover, stochastic switching persists under vdW interactions.

The relation between switching kinetics and steady-state occupation probabilities allows the identification of the critical parameter values for a first-order phase transition, where the switching between the two states occurs on the same timescales and the two states coexist on equal terms. This approach not only circumvents diagonalizing the Liouvillian but, more importantly, provides a criterion to distinguish bistability from metastability in finite systems.

The important distinction between metastability at the spectral and trajectory levels derives from two complimentary approaches to open quantum systems: the deterministic equation for the density matrix and the stochastic equation for the quantum state. While the evolution of density matrices is completely determined by initial conditions, stochastic quantum trajectories quickly converge into a metastable state. This leads to a hallmark of quantum bistability: the relaxation of quantum trajectories can slow down significantly compared with that of the corresponding density matrices.

Our analysis is not restricted to microscopic details and applies generally to homogeneous multistable systems. Given the enduring interest in the switching phenomenon and its potential applications, it is important to consider and exploit the exponential size scaling in quantum multistable systems. For example, by increasing (decreasing) the system size, signals with lower (higher) frequencies can be detected via stochastic resonance [47].

Finally, whether exponential size scaling also persists in large systems with finite-range interactions remains an open and important question. This warrants further investigation using scalable methods, such as adapted semi-classical [48,49] or tensor-network approaches [50].

## Supplementary Material

nwag146_Supplemental_File

## References

[1] Risken H, Vogel K. Quantum tunneling rates in dispersive optical bistability for low cavity damping. Phys Rev A 1988; 38: 1349–57. 10.1103/PhysRevA.38.1349 9900510

[2] Lapidus LJ, Enzer D, Gabrielse G. Stochastic phase switching of a parametrically driven electron in a Penning trap. Phys Rev Lett 1999; 83: 899–902. 10.1103/PhysRevLett.83.899

[3] Lee TE, Häffner H, Cross MC. Collective quantum jumps of Rydberg atoms. Phys Rev Lett 2012; 108: 023602. 10.1103/PhysRevLett.108.023602 22324684

[4] Fink JM, Dombi A, Vukics A et al. Observation of the photon-blockade breakdown phase transition. Phys Rev X 2017; 7: 011012. 10.1103/PhysRevX.7.011012

[5] Fink T, Schade A Höfling S et al. Signatures of a dissipative phase transition in photon correlation measurements. Nat Phys 2017; 14: 365–9. 10.1038/s41567-017-0020-9

[6] Ricci F, Rica RA, Spasenovic M et al. Optically levitated nanoparticle as a model system for stochastic bistable dynamics. Nat Commun 2017; 8: 15141. 10.1038/ncomms15141 28485372 PMC5436086

[7] Rondin L, Gieseler J, Ricci F et al. Direct measurement of Kramers turnover with a levitated nanoparticle. Nat Nanotechnol 2017; 12: 1130–3. 10.1038/nnano.2017.198 29209016

[8] Foss-Feig M, Niroula P, Young JT et al. Emergent equilibrium in many-body optical bistability. Phys Rev A 2017; 95: 043826. 10.1103/PhysRevA.95.043826 PMC6513354 31093586

[9] He J, Wang X, Wen X et al. Stochastic switching in the Rydberg atomic ensemble. Opt Express 2020; 28: 33682–9. 10.1364/OE.403689 33115027

[10] Mavrogordatos TK, Tancredi G, Elliott M et al. Simultaneous bistability of a qubit and resonator in circuit quantum electrodynamics. Phys Rev Lett 2017; 118: 040402. 10.1103/PhysRevLett.118.040402 28186805

[11] Minganti F, Bartolo N, Lolli J et al. Exact results for Schrödinger cats in driven-dissipative systems and their feedback control. Sci Rep 2016; 6: 26987. 10.1038/srep26987 27244292 PMC4886674

[12] Wu KD, Xie C, Li CF et al. Nonlinearity-enhanced continuous microwave detection based on stochastic resonance. Sci Adv 2024; 10: eado8130. 10.1126/sciadv.ado8130 39392887 PMC11639156

[13] Li H, Sun K, Yi W. Collective quantum stochastic resonance in Rydberg atoms. Phys Rev Res 2024; 6: L042046. 10.1103/PhysRevResearch.6.L042046

[14] Nigro D . On the uniqueness of the steady-state solution of the Lindblad–Gorini–Kossakowski–Sudarshan equation. J Stat Mech Theory Exp 2019; 2019: 043202. 10.1088/1742-5468/ab0c1c

[15] Minganti F, Biella A, Bartolo N et al. Spectral theory of Liouvillians for dissipative phase transitions. Phys Rev A 2018; 98: 042118. 10.1103/PhysRevA.98.042118

[16] Chen QM, Fischer M, Nojiri Y et al. Quantum behavior of the Duffing oscillator at the dissipative phase transition. Nat Commun 2023; 14: 2896. 10.1038/s41467-023-38217-x 37210421 PMC10199948

[17] Li Y, Zhou TG, Wu Z et al. Emergent universal quench dynamics in randomly interacting spin models. Nat Phys 2024; 20: 1966–72. 10.1038/s41567-024-02664-0

[18] Carr C, Ritter R, Wade CG et al. Nonequilibrium phase transition in a dilute Rydberg ensemble. Phys Rev Lett 2013; 111: 113901. 10.1103/PhysRevLett.111.113901 24074087

[19] Dykman MI, Mori E, Ross J et al. Large fluctuations and optimal paths in chemical kinetics. J Chem Phys 1994; 100: 5735–50. 10.1063/1.467139

[20] Zakine R, Vanden-Eijnden E. Minimum-action method for nonequilibrium phase transitions. Phys Rev X 2023; 13: 041044. 10.1103/PhysRevX.13.041044

[21] Touchette H. The large deviation approach to statistical mechanics. Phys Rep 2009; 478: 1–69. 10.1016/j.physrep.2009.05.002

[22] Letscher F, Thomas O, Niederprüm T et al. Bistability versus metastability in driven dissipative Rydberg gases. Phys Rev X 2017; 7: 021020. 10.1103/PhysRevX.7.021020

[23] Macieszczak K, Rose DC, Lesanovsky I et al. Theory of classical metastability in open quantum systems. Phys Rev Res 2021; 3: 033047. 10.1103/PhysRevResearch.3.033047

[24] Hänggi P, Talkner P, Borkovec M. Reaction-rate theory: fifty years after Kramers. Rev Mod Phys 1990; 62: 251–341. 10.1103/RevModPhys.62.251

[25] Lee TE, Häffner H, Cross MC. Antiferromagnetic phase transition in a nonequilibrium lattice of Rydberg atoms. Phys Rev A 2011; 84: 031402. 10.1103/PhysRevA.84.031402

[26] Browaeys A, Lahaye T. Many-body physics with individually controlled Rydberg atoms. Nat Phys 2020; 16: 132–42. 10.1038/s41567-019-0733-z

[27] Plenio MB, Knight PL. The quantum-jump approach to dissipative dynamics in quantum optics. Rev Mod Phys 1998; 70: 101–44. 10.1103/RevModPhys.70.101

[28] Helmrich S, Arias A, Lochead G et al. Signatures of self-organized criticality in an ultracold atomic gas. Nature 2020; 577: 481–6. 10.1038/s41586-019-1908-6 31942078

[29] Martin PC, Siggia ED, Rose HA. Statistical dynamics of classical systems. Phys Rev A 1973; 8: 423–37. 10.1103/PhysRevA.8.423

[30] Janssen HK . On a Lagrangean for classical field dynamics and renormalization group calculations of dynamical critical properties. Z Physik B 1976; 23: 377–80. 10.1007/BF01316547

[31] Ding DS, Busche H, Shi BS et al. Phase diagram and self-organizing dynamics in a thermal ensemble of strongly interacting Rydberg atoms. Phys Rev X 2020; 10: 021023. 10.1103/PhysRevX.10.021023

[32] Tan C, Lin X, Zhou Y et al. Dynamics of position-disordered Ising spins with a soft-core potential. Phys Rev B 2022; 105: 104204. 10.1103/PhysRevB.105.104204

[33] Weckesser P, Srakaew K, Blatz T et al. Realization of a Rydberg-dressed extended Bose-Hubbard model. Science 2025; 390: 849–53. 10.1126/science.adq7082 41264691

[34] Xu K, Sun ZH, Liu W et al. Probing dynamical phase transitions with a superconducting quantum simulator. Sci Adv 2020; 6: eaba4935. 10.1126/sciadv.aba4935 32596458 PMC7299620

[35] Pita-Vidal M, Wesdorp JJ, Andersen CK. Blueprint for all-to-all-connected superconducting spin qubits. PRX Quantum 2025; 6: 010308. 10.1103/PRXQuantum.6.010308

[36] Landig R, Hruby L, Dogra N et al. Quantum phases from competing short- and long-range interactions in an optical lattice. Nature 2016; 532: 476–9. 10.1038/nature17409 27064902

[37] Léonard J, Morales A, Zupancic P et al. Supersolid formation in a quantum gas breaking a continuous translational symmetry. Nature 2017; 543: 87–90. 10.1038/nature21067 28252072

[38] Helson V, Zwettler T, Mivehvar F et al. Density-wave ordering in a unitary Fermi gas with photon-mediated interactions. Nature 2023; 618: 716–20. 10.1038/s41586-023-06018-3 37225993 PMC10284702

[39] Macieszczak K, Guţă M, Lesanovsky I et al. Towards a theory of metastability in open quantum dynamics. Phys Rev Lett 2016; 116: 240404. 10.1103/PhysRevLett.116.240404 27367368

[40] Gardiner CW, Parkins AS, Zoller P. Wave-function quantum stochastic differential equations and quantum-jump simulation methods. Phys Rev A 1992; 46: 4363–81. 10.1103/PhysRevA.46.4363 9908637

[41] Dalibard J, Castin Y, Mølmer K. Wave-function approach to dissipative processes in quantum optics. Phys Rev Lett 1992; 68: 580–3. 10.1103/PhysRevLett.68.580 10045937

[42] Casteels W, Storme F, Le Boité A et al. Power laws in the dynamic hysteresis of quantum nonlinear photonic resonators. Phys Rev A 2016; 93: 033824. 10.1103/PhysRevA.93.033824

[43] Rodriguez SRK, Casteels W, Storme F et al. Probing a dissipative phase transition via dynamical optical hysteresis. Phys Rev Lett 2017; 118: 247402. 10.1103/PhysRevLett.118.247402 28665653

[44] Marcuzzi M, Buchhold M, Diehl S et al. Absorbing state phase transition with competing quantum and classical fluctuations. Phys Rev Lett 2016; 116: 245701. 10.1103/PhysRevLett.116.245701 27367395

[45] Coleman S. Quantum tunneling and negative eigenvalues. Nucl Phys B 1988; 298: 178–86. 10.1016/0550-3213(88)90308-2

[46] Elgart V, Kamenev A. Rare event statistics in reaction-diffusion systems. Phys Rev E 2004; 70: 041106. 10.1103/PhysRevE.70.041106 15600396

[47] Gammaitoni L, Hänggi P, Jung P et al. Stochastic resonance. Rev Mod Phys 1998; 70: 223–87. 10.1103/RevModPhys.70.223

[48] Singh VP, Weimer H. Driven-dissipative criticality within the discrete truncated Wigner approximation. Phys Rev Lett 2022; 128: 200602. 10.1103/PhysRevLett.128.200602 35657854

[49] Hosseinabadi H, Chelpanova O, Marino J. User-friendly truncated Wigner approximation for dissipative spin dynamics. PRX Quantum 2025; 6: 030344. 10.1103/1wwv-k7hg

[50] Sander A, Fröhlich M, Eigel M et al. Large-scale stochastic simulation of open quantum systems. Nat Commun 2025; 16: 11074. 10.1038/s41467-025-66846-x 41372191 PMC12698786

