# Supplementary material for: Switching metastable dynamics in many-body open quantum systems
Source: Natl Sci Rev. 2026 Mar 9;13(10):nwag146. doi: 10.1093/nsr/nwag146 (PMC13231515; doi:10.1093/nsr/nwag146)
Supplement: nwag146_Supplemental_File [file nwag146_supplemental_file.pdf]

Supplementary Material for
Switch Metastable Dynamics in Many-Body Open Quantum Systems

Ya-Xin Xiang,[1] Weibin Li,[2] Zhengyang Bai,[1,*] and Yu-Qiang Ma[1,3,†]

[1] *National Laboratory of Solid State Microstructures and School of Physics, Collaborative Innovation Center of Advanced Microstructures, Nanjing University, Nanjing 210093, China*

[2] *School of Physics and Astronomy, and Centre for the Mathematics and Theoretical Physics of Quantum Non-equilibrium Systems, University of Nottingham, Nottingham, NG7 2RD, UK*

[3] *Hefei National Laboratory, Hefei 230088, China*

In this supplementary material, we provide the interested reader with additional details on: the comparison of the heuristic and exact jump operators, the spectral analysis of the small-detuned monostable I regime; photon-emission (quantum-jump) statistics in the bistable and large-detuned monostable II regimes; trajectories from quantum-jump Monte-Carlo simulations; derivation of the Heisenberg-Langevin equations of motion and the Martin-Siggia-Rose construction; the instanton approach to stochastic switching, including the numerical scheme for solving the corresponding saddle-point equations; the connection and distinction between spectral and trajectory-level metastability, illustrated by the relation between spectral properties, switching rates and effective barriers; and metastability and switching dynamics in lattice systems with finite-range (van der Waals) interactions and local dissipation.

CONTENTS

I. Spectral decomposition 1
  A. Comparison of heuristic and exact jump operators 1
  B. Small-detuned monostable I regime 2

II. Bistability versus metastability 2
  A. Photon-emission statistics 2
  B. Finite-size scaling of occupation ratio 3

III. Trajectory dynamics 4
  A. Quantum-jump simulations 4
  B. Blinking-like large fluctuation 4
  C. Stochastic switching 5

IV. Heisenberg Langevin equation 5
  A. Equations of motion 5
  B. Quantum noise operators 5

V. Classical path integral 6
  A. Langevin equations 6
  B. The Martin-Siggia-Rose construction 6
  C. The instanton approach 7
  D. The gradient descent-ascent method for action minimization 8

VI. Spectral and trajectory-level metastability 8
  A. Approximate switching rate 8
  B. Effective barrier 9

VII. Results for Rydberg lattice 9
  A. All-to-all interaction 10
  B. van der Waals interaction 10

References 11

I. SPECTRAL DECOMPOSITION

A. Comparison of heuristic and exact jump operators

Before presenting results for the small-detuned monostable I regime, we provide a detailed analysis of the heuristic jump operator $\hat{L}$ introduced in Eq. (5) of the main text. Since the mapping of the coherent dynamics to the collective description is exact, we restrict our analysis to the dissipative part. Recall the conjugate master equation for an operator $\hat{X}$ under the action of $\hat{L}$ ($\gamma = 1$),

$$\partial_t \hat{X} = \hat{L}^\dagger \hat{X} \hat{L} - \frac{1}{2}\left\{\hat{L}^\dagger \hat{L}, \hat{X}\right\} \equiv \boldsymbol{\hat{D}}[\hat{X}, \hat{L}] \tag{I.1}$$

The resultant equations of motion for the operators $\hat{S}^-$ and $\hat{S}^z$ read ($N \gg 1$)

$$\partial_t \hat{S}^- = -\frac{1}{2}\sum_{M=-S}^{S} h_M^+ \left(1 - \frac{S+M}{S-M}\right) |M-1\rangle\langle M| \tag{I.2a}$$

$$= -\frac{1}{2}\hat{S}^- + \frac{1}{2}\sum_{M=-S}^{S}\left(\frac{S+M}{S-M}\right) h_M^+ |M-1\rangle\langle M|$$

$$\partial_t \hat{S}^z = -\sum_{M=-S}^{S}(M+S)|M\rangle\langle M| = -(\hat{S}^z + S\hat{I}) \tag{I.2b}$$

where $h_M^{\pm} = \sqrt{(S \mp M)(S \pm M + 1)}$.

* zhybai@nju.edu.cn
† myqiang@nju.edu.cn

Compared with the dissipative dynamics under the action of the original local dissipator $\hat{\sigma}_j^-$,

$$\partial_t \hat{S}^- = -\frac{1}{2}\hat{S}^- \tag{I.3a}$$

$$\partial_t \hat{S}^z = -(\hat{S}^z + S\hat{I}) \tag{I.3b}$$

The approximate collective description of local dissipation introduces an additional term,

$$\hat{\boldsymbol{D}}[\hat{X}, \hat{L}] - \hat{\boldsymbol{D}}\left[\hat{X}, \{\hat{\sigma}_l^-\}\right] = \sum_M \frac{h_M^+(S+M)}{2(S-M)} |M-1\rangle\langle M| \tag{I.4}$$

into the dynamics of quantum coherence $\hat{S}^{x,y}$. The effects of this additional term are more pronounced for states with higher excitation. For the dark state with $M \to -S$, this term is negligible. Nevertheless, this approximated jump operator enables systematic finite-size scaling. Results for the original (exact) local jump operators are presented in the last section [Sec. VII].

B. Small-detuned monostable I regime

In this section, we present the results of the spectral decomposition of the low-lying eigenmatrices of the Lindblad operator in the mean-field (MF) monostable I regime.

The eigenvalues and eigenmatrices of the Lindblad operator are obtained via solving $\hat{\mathcal{L}}\hat{\rho}_l = \lambda_l\hat{\rho}_l$, and sorted according to $\Re[\lambda_0] > \Re[\lambda_1] \geq ....$ As shown in Fig. 1. (a), for small detuning ($\Delta \lesssim 3$), the eigenvalues λ_1 and λ_2 are a pair of complex conjugates. The steady-state density operator $\hat{\rho}_{\rm ss}$ is the eigenmatrix $\hat{\rho}_0$ with the largest eigenvalue $\lambda_0 = 0$.

For excited states, the real parts of the eigenvalues satisfy $\Re[\lambda_l] \leq 0$. According to the spectral decomposition of density matrices [1], whether Hermitian or not, any eigenmatrix associated with a nonzero eigenvalue can be expressed as a linear combination of Hermitian operators with unit trace (i.e., density operators). For example, for a real eigenvalue λ_l, the corresponding eigenmatrix $\hat{\rho}_l$ is Hermitian and can be decomposed as follows

$$\hat{\rho}_l \propto \hat{\rho}_+ - \hat{\rho}_- \tag{I.5}$$

where $\hat{\rho}_+$ and $\hat{\rho}_-$ are mutually orthogonal density operators.

For a complex conjugate pair of eigenvalues λ_l and λ_l^* (satisfying $\hat{\mathcal{L}}\hat{\rho}_l^\dagger = \lambda_l^*\hat{\rho}_l^\dagger$), we can construct the two Hermitian combinations $\hat{p}_l = \hat{\rho}_l + \hat{\rho}_l^\dagger$ and $\hat{m}_l = i(\hat{\rho}_l^\dagger - \hat{\rho}_l)$ and arrive at

$$\hat{p}_l \propto \hat{\rho}_+ - \hat{\rho}_- \tag{I.6a}$$

$$\hat{m}_l \propto \hat{\phi}_+ - \hat{\phi}_- \tag{I.6b}$$

The expectation values of the excitation densities $\langle \hat{n}_e \rangle$ that are computed according to the density operators of

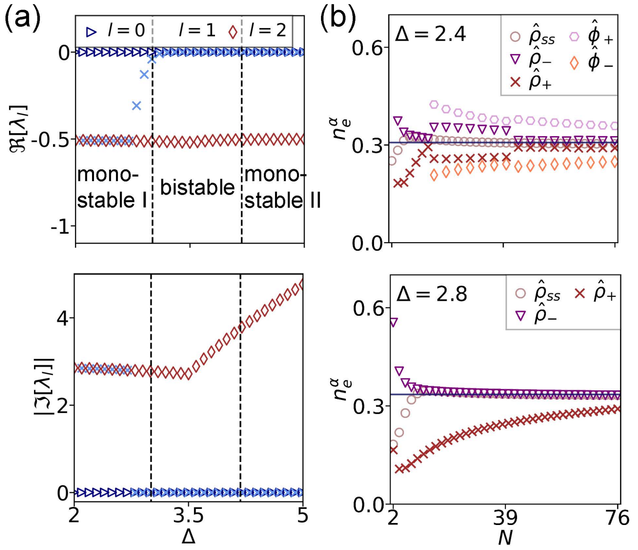

FIG. 1. (a) The real (upper) and imaginary (lower) parts of the first three eigenvalues of the Lindblad operator for $N = 54$, obtained via solving $\hat{\mathcal{L}}\hat{\rho} = \lambda\hat{\rho}$. The eigenvalues are sorted by their real parts in descending order, $\Re[\lambda_0] > \Re[\lambda_1] \geq ....$ The mean-field (MF) bistable regime is located between the two black dashed lines. (b) The excitation densities according to the steady-state density operator $\hat{\rho}_{\rm ss}$ and the density matrices from the spectral decomposition of the eigenmatrices associated with the eigenvalues that have the second largest real part. The blue lines represent the MF excitation densities.

the lowest excited state $\hat{\rho}_{+(-)}$ and $\hat{\phi}_{+(-)}$, as well as the steady-state density operator $\hat{\rho}_{\rm ss}$ are plotted in Fig. 1(b). The steady-state excitation densities are found to agree with the MF values.

For $\Delta = 2.4$, the presence of a complex conjugate pair of eigenvalues λ_1 and $\lambda_2 = \lambda_1^*$ [see Fig. 1(a)] results in four density operators with excitation densities scattering around the MF value.

Upon increasing Δ to 2.8, close to the onset of bistability, the eigenvalue λ_1 becomes real, and the resultant two density operators $\hat{\rho}_+$ and $\hat{\rho}_-$ give rise to two metastable states with high and low excitation densities. In contrast to the bistable regime, where the excitation densities of the two subspaces are related to those of the two MF states, the difference in the excitation densities between $\hat{\rho}_+$ and $\hat{\rho}_-$ vanishes with increasing system sizes.

II. BISTABILITY VERSUS METASTABILITY

A. Photon-emission statistics

Signatures of discontinuous dissipative phase transitions in finite systems also manifest in the photon-emission (quantum-jump) statistics, as trajectories in different metastable states exhibit different collapse and phonton emission rates [2–4].

In the long-time limit $t \gg 1$, the probability $P_t(K)$ of observing K emitted photons adopts a large-deviation (LD) form [5]

$$P_t(K) \approx e^{-t\phi(k)} \tag{II.1}$$

with $k = K/t$ being the time-averaged photon emission rate.

The LD function $\phi(k)$ contains all information about the probability of K at long times. The statistics of K is described via the generating function, which also acquires a LD form, i.e.,

$$Z_t(s) \equiv \sum_{K=0}^{\infty} P_t(K)e^{-sK} \approx e^{t\theta(s)} \tag{II.2}$$

Here s is the conjugate field to the dynamic observable K, and the two LD functions $\theta(s), \phi(k)$ are related by a Legendre transform [5, 6],

$$\theta(s) = -\min_k [\phi(k) + ks] \tag{II.3}$$

We obtain the LD function $\theta(s)$ by finding directly the largest eigenvalue of the tilted generator [6–9],

$$\hat{\mathcal{L}}_s[\hat\rho] = i[\hat\rho, \hat H_{\text{eff}}] + e^{-s}\hat L \hat\rho \hat L^\dagger \tag{II.4}$$

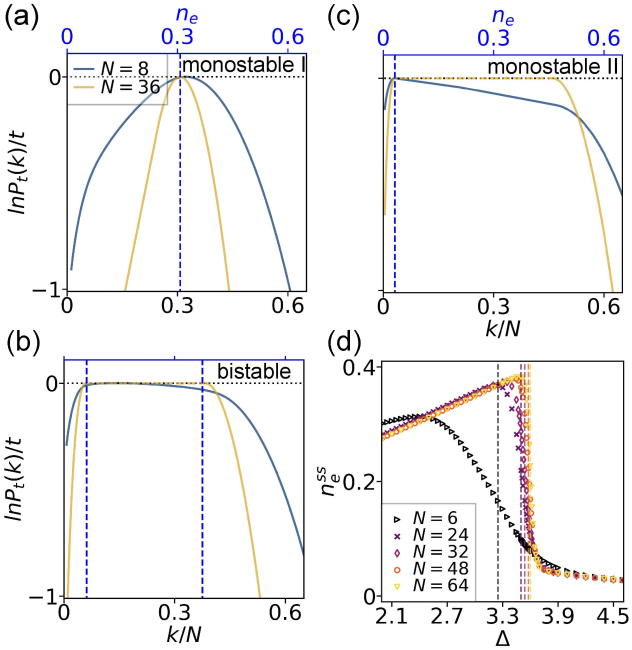

FIG. 2. Signatures of first-order phase transition in finite systems. Statistics of time-averaged photon emission rate in systems with different number of atoms (color-coded) obtained within the quantum large-deviation (LD) formalism for (a) $\Delta = 2.4$, (b) $\Delta = 3.4$, and (c) $\Delta = 4.4$. The rise of two maxima connected by vertical dashed lines indicates kink of the LD functions. (d) The steady-state excitation population $n_e^{\text{ss}} = \text{Tr}[\hat n_e \hat\rho_{\text{ss}}]$ as a function of detuning Δ for different system sizes N. The blue dashed lines in (a)-(c) represent the excitation population of the MF stable fixed points. The vertical dashed lines in (d) stand for the maximum of $|\partial_\Delta n_e^{\text{ss}}|$ for a given system size N (color-coded).

where the heuristic jump operator in terms of collective Dicke states $|M\rangle$ [10] is given by

$$\hat L = \sum_M \sqrt{M+S}\, |M-1\rangle \langle M| \tag{II.5}$$

and the effective non-Hermitian Hamiltonian $\hat H_{\text{eff}}$ takes the forms

$$\hat H_{\text{eff}} = \Omega \hat S^x - \frac{V}{2N}\hat S^+ \hat S^- + \left(\frac{V - i\gamma}{2} - \Delta\right)\hat S^z - i\frac{S}{2} \tag{II.6}$$

with the operators $\hat S^\pm = \sum_l \hat\sigma_l^\pm$ satisfying

$$\hat S^\pm |M\rangle = \sqrt{(S \mp M)(S \pm M + 1)}\, |M \pm 1\rangle \tag{II.7}$$

and the commutation relation

$$[\hat S^+, \hat S^-] = 2\hat S^z \tag{II.8}$$

B. Finite-size scaling of occupation ratio

Because photon emission is attributed to the spontaneous decay of excited atoms, at late times $t \gg 1$, the average number of emitted photons approaches the steady-state excitation population, i.e. $\langle K\rangle/t \approx N n_e$. As shown in Figs. 2(a) and (b), in bistable regime [$\Delta = 3.4$; see panel (a)], the LD function

$$-\phi(k) \equiv \lim_{t\to+\infty} \frac{1}{t}\ln P_t(K) \tag{II.9}$$

is bimodal, vanishing completely between two maxima, corresponding to high and low excitation populations. This originates from singularities (kinks) of the LD function $\theta(s)$ and is indicative of distinct dynamical phases [5, 7, 8, 11, 12]. Also, the steady-state excitation population n_e^{ss} is a mixture of the dark and bright states and asymptotically approaches the bright (dark) state for small (large) detuning when $N \to \infty$ [see Fig. 2(d)]. This asymptotic behavior leads to a discontinuous jump in n_e^{ss} at a critical detuning Δ_c in the thermodynamic limit.

The kinks in the LD functions and the concomitant bimodal statistics persist even after further increasing the detuning to $\Delta = 4.4$ [monostable II, see panel (b)], which is at odds with MF predictions. The discrepancy is attributed to the emergence of a diverging relaxation time—metastability in the absence of bistability—which also induces singularities in the LD functions [8, 13]. This metastability stems from a real λ_1 and the associated metastable states $\hat\rho_\pm$.

As shown in Figs. 3(a) and (b), the occupation ratio r serves as a key diagnostic to distinguish the bistable and monostable II regimes. Although both regimes share similar spectral properties, their occupation statistics differ greatly. In the bistable regime, the ratio r varies exponentially with system size N, with a detuning-dependent exponent. In contrast, monostable II regime deviate

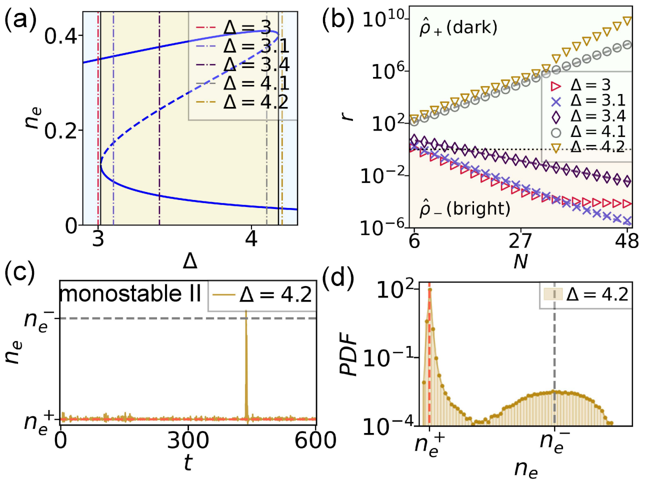

FIG. 3. Comparison of metastability in bistable and monostable regimes. (a) Mean-field phase diagram. Stable (blue solid) and unstable (blue dashed) fixed points as a function of detuning Δ. Bistable is shaded in yellow; monostable regimes I and II are shaded in blue. Phase boundaries are indicated by black vertical lines. Selected detuning values are marked by color-coded vertical dashdotted lines. (b) Finite-size scaling of the ratio $r = D[\hat{\rho}_+, \hat{\rho}_{\rm ss}]/D[\hat{\rho}_-, \hat{\rho}_{\rm ss}]$ of the steady-state occupation probabilities of the quantum dark ($\hat{\rho}_+$) and bright ($\hat{\rho}_-$) states in monostable I ($\Delta = 3$), bistable ($\Delta = 3.1, 3.4, 4.1$) and monostable II ($\Delta = 4.2$) regimes. (c) Individual quantum trajectory and (d) probability distribution function (PDF) of the Rydberg population in the monostable II regime for $\Delta = 4.2, N = 12$. The red and gray dashed lines mark the Rydberg densities $n_e^{\pm}$ of $\hat{\rho}_{\pm}$.

strongly from this exponential scaling. It is precisely this exponential scaling of the occupations, in addition to the spectral metastability, that gives rise to the Arrhenius law governing stochastic switching in the bistable regime, as discussed in the following section.

III. TRAJECTORY DYNAMICS

A. Quantum-jump simulations

In this section, we discuss the long-time dynamics of the system by means of quantum-jump simulations. In the simulation, given the time step dt, at each step the wave function $|\psi_t\rangle$ either collapses

$$|\psi_t\rangle \to \frac{\hat{L}\,|\psi_t\rangle}{\sqrt{\langle\psi_t|\,\hat{L}^\dagger\hat{L}\,|\psi_t\rangle}} \tag{III.1}$$

with a probability of $P_t = dt\,\langle\psi_t|\,\hat{L}^\dagger\hat{L}\,|\psi_t\rangle$, or evolves under the action of the effective non-Hermitian Hamiltonian (II.6)

$$|\psi_t\rangle \to \frac{e^{-i\hat{H}_{\rm eff}dt}\,|\psi_t\rangle}{\sqrt{1-P_t}} \tag{III.2}$$

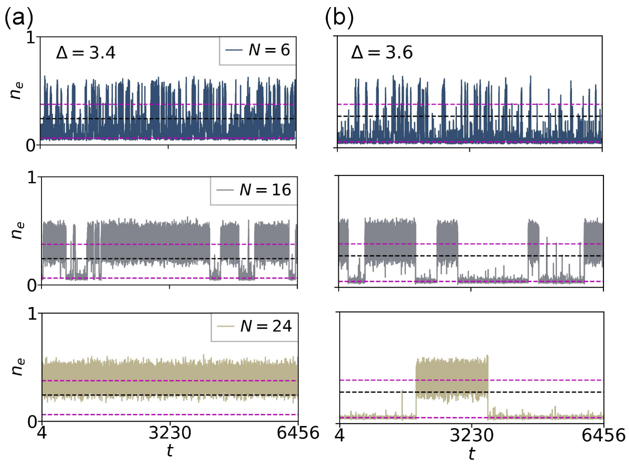

FIG. 4. Average excitation population of simulated quantum trajectory for (a) $\Delta = 3.4$ and (b) $\Delta = 3.6$ of varying number of atoms N over time. The gray (magenta) dashed lines denote the unstable (stable) MF fixed point(s).

with a probability $1 - P_t$. The time-dependent average Rydberg population is computed according to [2–4]

$$n_e(t) = \frac{1}{N}\,\langle\psi_t|\,\hat{S}^z\,|\psi_t\rangle + \frac{1}{2} \tag{III.3}$$

B. Blinking-like large fluctuation

Despite the absence of true bistability, the presence of a large real λ_1 in the monostable II regime does have a dynamical signature. Trajectories in this regime can display two distinct typical photon emission rates $n_e^{\pm}$, which can be directly attributed to the two quantum metastable states $\hat{\rho}_{\pm}$. As can be seen from Fig. 3(c), an individual trajectory exhibits blinking-like fluctuations: the system resides predominantly in the dark state, only briefly visiting the bright state during a rare deviation before returning to the dark state for a significantly long dwelling time.

Consequently, the probability distribution function (PDF) of the Rydberg density, shown in Fig. 3(d), reveals a bimodal distribution, even though the occupation probability of the dark state is four orders of magnitude larger than that of the bright state. A similar effect—spectral metastability leading to blinking-like dynamics— is observed in a monostable three-level single-atom system [13].

Therefore, in the monostable II regime, the quantum bright state might act like a dynamical saddle point: while the dynamics might approach it in certain directions, the state itself is unstable, and the system will subsequently be driven away. This blinking-like dynamics differs fundamentally from stochastic switching between two metastable states within the bistable regime.

C. Stochastic switching

In what follows, we refer to the quantum switching from the dark (bright) to bright (dark) states as upward (downward). By keeping track of the upward and downward switching from these simulated trajectories, we extract the corresponding switching times. Exemplary quantum trajectories are displayed in Fig. 4, where it is evident that for small N, the system is mainly trapped in a dark state and occasionally experiences significant quantum fluctuations from time to time (upper panels). In contrast, the wave function jumps back and forth between the two states (central panels) in larger systems. Relative to the time spent trapped around either of the two states, the jump appears almost instantaneously, and the waiting times between successive jumps increase with N, indicating elongated lifetimes of the two states. This is consistent with the smaller gap of the Liouvillian spectrum. Additionally, in larger systems, as Δ increases from 3.4 to 3.6, the waiting times for upward jumps surpass those for downward jumps [central and lower panels in Fig. 4(a) and (b)]. This suggests that the dark (bright) state is more stable than the other for large (small) detuning in the bistable region, consistent with the spectral decomposition results shown in the main text.

IV. HEISENBERG LANGEVIN EQUATION

A. Equations of motion

We use the stochastic variant of the conjugate master equation for an observable $\hat{X}$ [14]($\hbar = 1$),

$$\partial_t \hat{X} = i[\hat{H}, \hat{X}] + \sum_k \left(\hat{L}_k^\dagger \hat{X} \hat{L}_k - \frac{1}{2}\{\hat{L}_k^\dagger L_k, \hat{X}\} + \hat{\xi}_k^X \right) \tag{IV.1}$$

where $\hat{L}_k = \hat{\sigma}_k^-$ is the dissipator, and $\hat{\xi}_k^X$ is the corresponding noise operator, and the Hamiltonian

$$\hat{H} = \sum_l \left[-\Delta \hat{n}_l + \Omega \hat{\sigma}_l^x + \frac{V}{N-1} \sum_{l<k} \hat{n}_k \hat{n}_l \right] \tag{IV.2}$$

where $\hat{\sigma}_l^\alpha$ ($\alpha = x, y, z$) denote the Pauli matrices acting on the l-th atom, and the operators $\hat{\sigma}_k^\pm \equiv \hat{\sigma}_k^x \pm \hat{\sigma}_k^y$ flip the atomic state, and $\hat{n}_l = \hat{\sigma}_l^z + \mathbf{I}_l/2$ represents the Rydberg number operator. The last term of the Hamiltonian (IV.2) represents the Rydberg-Rydberg interactions between the l-th and k-th atoms after a MF approximation.

In what follows, we use γ^{-1} as the time unit. The resultant Heisenberg-Langevin equations of motion read

$$\partial_t \hat{\sigma}_k^x = \Delta \hat{\sigma}_k^y - \frac{V \sum_l (\hat{\sigma}_k^y \hat{n}_l + \hat{n}_l \hat{\sigma}_k^y)}{2(N-1)} - \frac{1}{2}\hat{\sigma}_k^x + \hat{\xi}_k^x \tag{IV.3a}$$

$$\partial_t \hat{\sigma}_k^y = -\Omega \hat{\sigma}^z - \frac{1}{2}\hat{\sigma}_k^y + \frac{V \sum_l (\hat{\sigma}_k^x \hat{n}_l + \hat{n}_l \hat{\sigma}_k^x)}{2(N-1)} - \Delta \hat{\sigma}_k^x + \hat{\xi}_k^y \tag{IV.3b}$$

$$\partial_t \hat{\sigma}_k^z = \Omega \hat{\sigma}_k^y - \hat{n}_k + \hat{\xi}_k^z \tag{IV.3c}$$

B. Quantum noise operators

We follow [14, 15] to write the local Hamiltonian $\hat{H}_d$ to represent the spontaneous decay of excited atoms due to coupling to a reservoir (atomic indices are omitted hereinafter)

$$\hat{H}_d = \sum_q \kappa_q \left(\hat{\sigma}^+ \hat{d}_q + \hat{d}_q^\dagger \hat{\sigma}^- \right) + \sum_q \omega_q \hat{d}_q^\dagger \hat{d}_q \tag{IV.4}$$

where the operators $\hat{d}_q, \hat{d}_q^\dagger$ are local bosonic bath modes satisfying $[\hat{d}_q, \hat{d}_{q'}^\dagger] = \delta_{q,q'}$ with their respective frequencies ω_q and the coupling strength κ_q.

The Heisenberg equations under the action of $\hat{H}_d$ read

$$\partial_t \hat{\sigma}^z = i\left[\hat{H}_d, \hat{\sigma}^z\right] = i \sum_q \kappa_q \left(\hat{d}_q^\dagger \hat{\sigma}^- - \hat{\sigma}^+ \hat{d}_q \right) \tag{IV.5a}$$

$$\partial_t \hat{\sigma}^+ = i\left[\hat{H}_d, \hat{\sigma}^+\right] = -2i \sum_q \kappa_q \hat{d}_q^\dagger \hat{\sigma}^z \tag{IV.5b}$$

$$\partial_t \hat{d}_q = i\left[\hat{H}_d, \hat{d}_q\right] = -i\kappa_q \hat{\sigma}^- - i\omega_q \hat{d}_q \tag{IV.5c}$$

Formally integration of the last equation (IV.5c) leads to

$$\hat{d}_q(t) = \hat{d}_q(0) e^{-i\omega_q t} - i\kappa_q \int_0^t dt' \hat{\sigma}^-(t') e^{-i\omega_q (t-t')} \tag{IV.6}$$

Plugging it into the the first two equations yields

$$\partial_t \hat{\sigma}^z = i \sum_q \kappa_q \left[\hat{d}_q^\dagger(0) \hat{\sigma}^-(t) e^{i\omega_q t} - \text{H.c.} \right] - \sum_q \kappa_q^2 \int_0^t dt' \left[\hat{\sigma}^+(t') \hat{\sigma}^-(t) e^{i\omega_q (t-t')} + \text{H.c.} \right] \tag{IV.7a}$$

$$\partial_t \hat{\sigma}^+ = -2i \sum_q \kappa_q \hat{d}_q^\dagger(0) \hat{\sigma}^z(t) e^{i\omega_q t} + 2\sum_q \kappa_q^2 \int_0^t dt' \hat{\sigma}^+(t') \hat{\sigma}^z(t) e^{i\omega_q (t-t')} \tag{IV.7b}$$

where H.c. is the shorthand notation for Hermitian conjugate.

The first summations on the right hand side of Eqs. (IV.7a) and (IV.7b) are the noise operators correspond-

ing to the spontaneous decay process, i.e.,

$$\hat{\xi}^z(t) = i\sum_q \kappa_q \left[\hat{d}_q^\dagger(0)\hat{\sigma}^-(t)e^{i\omega_q t} - \text{H.c.}\right] \quad \text{(IV.8a)}$$

$$\hat{\xi}^+(t) = -2i\sum_q \kappa_q \hat{d}_q^\dagger(0)\hat{\sigma}^z(t)e^{i\omega_q t} \quad \text{(IV.8b)}$$

and the second summations represent the dissipation.

In the Weisskopf-Wigner approximation, the summation over yields a delta function $\delta(t-t')$ [16], and the integration within the dissipation terms can be carried out. As a results, the dissipation rate is related to the effective coupling constant $\kappa(0)$ and the bath density of states evaluated at zero frequency $D(0) = \sum_q \delta(\omega_q)$, via $2\pi D(0)[\kappa(0)]^2 = 1$.

We further suppose that the reservoir is in thermal equilibrium and kept at zero temperature, described by the density operator $\hat{\rho}_B = |0\rangle\langle 0|$, where $|0\rangle$ is the vacuum state. It follows that the average number of bath bosons $n_q = \langle \hat{d}_q^\dagger \hat{d}_q\rangle \equiv \text{Tr}\,[\hat{d}_q^\dagger \hat{d}_q \hat{\rho}_B] = 0$, and that the noise operators have zero mean $\langle \hat{\xi}^{z/+}\rangle = 0$. However, the covariance is not necessarily zero, and the non-vanishing one includes

$$\langle \hat{\xi}^z(t)\hat{\xi}^z(t')\rangle = \hat{n}\delta(t-t') \quad \text{(IV.9a)}$$

$$\langle \hat{\xi}^z(t)\hat{\xi}^+(t')\rangle = \hat{\sigma}^+\delta(t-t') \quad \text{(IV.9b)}$$

$$\langle \hat{\xi}^-(t)\hat{\xi}^+(t')\rangle = \delta(t-t') \quad \text{(IV.9c)}$$

Together with $\hat{\xi}^x = \frac{1}{2}(\hat{\xi}^+ + \hat{\xi}^-), \hat{\xi}^y = \frac{i}{2}(\hat{\xi}^- - \hat{\xi}^+)$, one arrives at

$$\langle \hat{\xi}^z(t)\hat{\xi}^x(t')\rangle = \frac{1}{2}\hat{\sigma}^+\delta(t-t') \quad \text{(IV.10a)}$$

$$\langle \hat{\xi}^z(t)\hat{\xi}^y(t')\rangle = -\frac{i}{2}\hat{\sigma}^+\delta(t-t') \quad \text{(IV.10b)}$$

$$\langle \hat{\xi}^x(t)\hat{\xi}^y(t')\rangle = \langle \hat{\xi}^y(t)\hat{\xi}^x(t')\rangle = -\frac{i}{4}\delta(t-t') \quad \text{(IV.10c)}$$

$$\langle \hat{\xi}^x(t)\hat{\xi}^x(t')\rangle = \langle \hat{\xi}^y(t)\hat{\xi}^y(t')\rangle = \frac{1}{4}\delta(t-t') \quad \text{(IV.10d)}$$

The covariance $(\alpha, \beta = x, y, z)$ $C_{\alpha\beta}$ of classical noises ξ^α, ξ^β is extracted from the Hermitian part of the corresponding quantum noise covariance [14] $1/2(\langle \hat{\xi}^\alpha \hat{\xi}^\beta\rangle +$ H.c.).

Introducing the vectorial notation

$$\boldsymbol{\xi} = \begin{pmatrix}\xi^x, & \xi^y, & \xi^z\end{pmatrix}^T \quad \text{(IV.11)}$$

we can write the classical noise covariance in a compact form $\langle \boldsymbol{\xi}_l(t)\boldsymbol{\xi}_k^T(t')\rangle = \delta_{l,k}\delta(t-t')\boldsymbol{C}$ with

$$\boldsymbol{C} = \frac{1}{2}\begin{pmatrix}\frac{1}{2} & 0 & \sigma^x \\ 0 & \frac{1}{2} & \sigma^y \\ \sigma^x & \sigma^y & 2n\end{pmatrix} \quad \text{(IV.12)}$$

where $(\alpha = x, y, z)$ $\sigma^\alpha = \langle \sigma^\alpha\rangle$ and $n \equiv \langle \hat{n}\rangle$.

V. CLASSICAL PATH INTEGRAL

A. Langevin equations

Introducing the collective spin operators $(\alpha = x, y, z)$ $\hat{S}^\alpha = \sum_l \hat{\sigma}_l^\alpha$, after omitting the operator moments generated by the many-body interactions, we obtain the following set of coupled nonlinear equations for the expectation values $m_\alpha \equiv \text{Tr}\,[\hat{S}^\alpha\hat{\rho}]/S$ (with the total spin $S = N/2$)

$$\partial_t m_x = -\left(Vn_e - \Delta\right)m_y - \frac{1}{2}m_x + \eta_x \quad \text{(V.1a)}$$

$$\partial_t m_y = -\Omega m_z + \left(Vn_e - \Delta\right)m_x - \frac{1}{2}m_y + \eta_y \quad \text{(V.1b)}$$

$$\partial_t m_z = \Omega m_y - (m_z + 1) + \eta_z \quad \text{(V.1c)}$$

where the Rydberg densities

$$n_e \equiv \frac{1}{N}\text{Tr}\left[\sum_l \hat{n}_l\hat{\rho}\right] = \frac{1}{2}(m^z + 1) \quad \text{(V.2)}$$

and η_x, η_y, η_z are Markovian white noises defined as $\eta_\alpha = 1/S\sum_l \xi_l^\alpha$ with covariance

$$M_{\alpha\beta} \equiv \langle \eta_\alpha\eta_\beta\rangle = \frac{\sum_{kl}\langle \xi_l^\alpha \xi_k^\beta\rangle}{S^2} = \frac{2C_{\alpha\beta}}{S} \quad \text{(V.3)}$$

Note that because the noises are Markovian, noise covariance between different atoms vanishes, and the local correlator $C_{\alpha\beta}$ has no spatial dependence.

Introducing the vectorial notation,

$$\boldsymbol{\eta} = \begin{pmatrix}\eta_x, & \eta_y, & \eta_z\end{pmatrix}^T \quad \text{(V.4)}$$

the covariance $\langle \boldsymbol{\eta}_l(t)\boldsymbol{\eta}_k^T(t')\rangle = \boldsymbol{M}\delta(t-t')\delta_{k,l}$ can be explicitly written,

$$\boldsymbol{M} = \frac{1}{N}\begin{pmatrix}1 & 0 & m_x \\ 0 & 1 & m_y \\ m_x & m_y & 2(m_z+1)\end{pmatrix} \quad \text{(V.5)}$$

Also, by use of (V.2), the covariance between the noise of Rydberg densities η_n and others, e.g., $\langle \eta_n\eta_{x,y}\rangle$ as well as $\langle \eta_n^2\rangle$ can by computed via the relation $\eta_n = \eta_z/2$.

B. The Martin-Siggia-Rose construction

From the Langevin equations (V.1), one can construct a functional integration representation in terms of the Janssen-de Dominicis formalism [17], by introducing an auxiliary Martin-Siggia-Rose response field [18].

To be specific, given the initial condition $\boldsymbol{m}(t)$, the presence of Langevin noises $\boldsymbol{\eta}$ leads to a conditional probabilities for the final state $\boldsymbol{m}'$ after a short period of time dt, viz.

$$P_{\boldsymbol{m}\to\boldsymbol{m}'} = \int d\boldsymbol{\eta}\frac{e^{-\frac{dt}{2}\boldsymbol{\eta}\cdot\boldsymbol{M}^{-1}\cdot\boldsymbol{\eta}^T}\delta[(\partial_t\boldsymbol{m} - \boldsymbol{\eta} - \boldsymbol{F})dt]}{\sqrt{(2\pi)^3\det[dt\boldsymbol{M}]}} \quad \text{(V.6)}$$

where $\partial_t \boldsymbol{m} = \frac{1}{dt}(\boldsymbol{m}' - \boldsymbol{m})$ and $\boldsymbol{F}$ are the deterministic parts of Eqs. (V.1). Plugging in the Fourier transform of the delta function

$$\delta(\boldsymbol{r}) = \int \frac{d\tilde{\boldsymbol{r}} e^{-\tilde{\boldsymbol{r}}\cdot\boldsymbol{r}}}{(2\pi)^3} \tag{V.7}$$

followed by integrating out $\boldsymbol{\eta}$, we arrive at

$$P_{\boldsymbol{m}\to\boldsymbol{m}'} = \int d\tilde{\boldsymbol{m}} e^{-\tilde{\boldsymbol{m}}\cdot\left(\partial_t \boldsymbol{m} - \boldsymbol{F} - \frac{1}{2}\boldsymbol{M}\cdot\tilde{\boldsymbol{m}}^T\right)} \tag{V.8}$$

The transition probability during a finite time follows from summation of all trajectories with fixed initial and ending points, giving rise to a Gaussian functional, which is also the desired path integral

$$\mathcal{Z} = \int \mathcal{D}[\boldsymbol{m}, \tilde{\boldsymbol{m}}] e^{-\mathcal{S}} \tag{V.9}$$

where the action

$$\mathcal{S} = \int dt \tilde{\boldsymbol{m}} \cdot \left(\partial_t \boldsymbol{m} - \boldsymbol{F} - \frac{1}{2}\boldsymbol{M}\cdot\tilde{\boldsymbol{m}}^T\right) \tag{V.10}$$

and the imaginary fields $\tilde{\boldsymbol{m}}$ are referred to as the response fields (also called the Martin-Siggia-Rose auxiliary fields) conjugate to $\boldsymbol{m}$ and are related to the dynamic responses of $\boldsymbol{m}$ to perturbations.

C. The instanton approach

We begin by noting that integrating out the response fields $\tilde{\boldsymbol{m}}$, the action (V.10) becomes

$$\mathcal{S} = \int \frac{dt}{2}(\partial_t \boldsymbol{m} - \boldsymbol{F}) \cdot \boldsymbol{M}^{-1} \cdot (\partial_t \boldsymbol{m} - \boldsymbol{F})^T \geq 0 \tag{V.11}$$

which vanishes along the deterministic, noiseless paths $\partial_t \boldsymbol{m} = \boldsymbol{F}$. In contrast, escaping a fixed point driven by noises entails deviations from the deterministic paths, which in turn results in nonzero action.

Among all possible escaping paths connecting two distinct fixed points, the instanton (optimal path) is the one with the minimal action. Variation of the action (V.10) with respect to $(\alpha = x, y, z)$ $m_\alpha, \tilde{m}_\alpha$ leads to the following saddle-point equations,

$$\partial_t m_\alpha = F_\alpha + M_{\alpha\beta}\tilde{m}_\beta \tag{V.12a}$$

$$\partial_t \tilde{m}_\alpha = -\partial_{m_\alpha} F_\beta \tilde{m}_\beta - \frac{1}{2}\tilde{m}_\beta \partial_{m_\alpha} M_{\beta\nu}\tilde{m}_\nu \tag{V.12b}$$

where the Einstein's convention of summing up the repeated indices is made implicit.

We can immediately identify the MF fixed points that nullify $\boldsymbol{F}$ and with zero noises $\boldsymbol{m}$ as fixed points of the above equations. Let $\boldsymbol{J}$ be the Jacobian of the MF Eqs. (V.1), then the Jacobian $\tilde{\boldsymbol{J}}$ of the expanded six-dimensional saddle-point equations is given by

$$\tilde{\boldsymbol{J}} = \begin{pmatrix} \boldsymbol{J} & \boldsymbol{M} \\ \boldsymbol{0} & -\boldsymbol{J} \end{pmatrix} \tag{V.13}$$

whose eigenvalues λ are obtained from the eigenfunctions

$$0 = \det\left[\tilde{\boldsymbol{J}} - \lambda \boldsymbol{I}\right] = \det\left[\boldsymbol{J} - \lambda\boldsymbol{I}\right]\det\left[-\boldsymbol{J} - \lambda\boldsymbol{I}\right] \tag{V.14}$$

It is evident that the MF stable fixed points become unstable fixed points of the saddle-point equations (V.12). This implies that all stable fixed points are metastable in the presence of noises, which also raises a technical issue: direct iteration leads to numerical instabilities. To address this, the gradient descent-ascent (GDA) method [19], which will be detailed below, can be employed.

Inserting the saddle-point equations (V.12) to the action (V.10), we arrive at the minimized action

$$\mathcal{S}_{T,E} = \frac{1}{2}\int_0^T dt \tilde{m}_\alpha M_{\alpha\beta}\tilde{m}_\beta \tag{V.15}$$

where the subscripts "energy" E and evolution time T specify the path.

Introducing the momenta $q_\alpha = \tilde{m}_\alpha / N$, Eqs. (V.12) can be cast into Hamilton-Jacobi equations,

$$\partial_t m_\alpha = \partial_{q_\alpha} H \tag{V.16a}$$

$$\partial_t q_\alpha = -\partial_{m_\alpha} H \tag{V.16b}$$

with the classical Hamiltonian

$$H = q_\alpha F_\alpha + \frac{1}{2} q_\alpha \bar{M}_{\alpha\beta} q_\beta \tag{V.17}$$

and the rescaled covariance matrix $\bar{\boldsymbol{M}} = N\boldsymbol{M}$.

The Hamiltonian (V.17) is an integral of motion, i.e., $H = E$. The energy E is determined by the time boundary conditions and vanishes for MF fixed points ($\tilde{\boldsymbol{m}} = 0$). Therefore, the minimized action (V.15) becomes

$$\begin{aligned}\mathcal{S}_{T,E} &= N\int_0^T dt(q_\alpha \partial_t m_\alpha - H) \\ &= N\left(\int_0^T q_\alpha dm_\alpha - ET\right) \\ &= N\int_0^T q_\alpha dm_\alpha\end{aligned} \tag{V.18}$$

This suggests as we minimize the action (V.10) according to the Hamilton-Jaccobi equations (V.16) with both initial and final points being the MF fixed points, we will arrive at a zero-energy path (also known as the optimal path [20]).

The energy barrier (quasipotential) $\Phi(\boldsymbol{m}_a \to \boldsymbol{m}_b)$ from state $\boldsymbol{m}_a$ to $\boldsymbol{m}_b$ is defined as the accumulated action through the optimal path [6, 19–27]

$$\Phi(\boldsymbol{x}_a \to \boldsymbol{x}_b) \equiv \inf_{T>0} \inf \mathcal{S}_{T,E} \tag{V.19}$$

which is also therefore the minimized action (V.18) and is thus independent of time T.

It is worth noting that the evolution time T is determined self-consistently by the path, and it does not influence the results provided that it is sufficiently long. Assume that we have obtained an path $\boldsymbol{x}(t) = \big(\boldsymbol{m}(t),\ \boldsymbol{q}(t)\big)$ connecting the initial state $\boldsymbol{x}_i$ to the final state $\boldsymbol{x}_f$, then the evolution time is given by

$$T = \int_{\boldsymbol{x}_a}^{\boldsymbol{x}_b} \frac{|d\boldsymbol{x}|}{|\partial_t \boldsymbol{x}|} \tag{V.20}$$

Because remaining in any fixed points for arbitrarily long times does not lead to any action increment, we can treat T as a free parameter and increase T until the action converges.

D. The gradient descent-ascent method for action minimization

As mentioned earlier, direct integration of the Hamilton-Jacobi equations (V.16) is numerically unstable. To address this, we resort to GDA method that translates the minimization of action (V.19) for a given T into a constrained min-max problem [19], i.e.,

$$\inf \mathcal{S}_{T,E} = N \max_{\boldsymbol{q}} \min_{\boldsymbol{m}} \mathcal{A}_T[\boldsymbol{m}, \boldsymbol{q}] \tag{V.21}$$

where the functional

$$\mathcal{A}_T[\boldsymbol{m}, \boldsymbol{q}] = \int_0^T dt (q_\alpha \partial_t m_\alpha - H) \tag{V.22}$$

where the Hamiltonian H is given by Eq. (V.17).

This GDA method leads to the following evolution equations (in optimization time τ)

$$\partial_\tau m_\alpha = -\nu_\alpha \frac{\delta \mathcal{A}_T}{\delta m_\alpha} = \nu_\alpha \left(\partial_t q_\alpha + \partial_{m_\alpha} H\right) \tag{V.23a}$$

$$\nu_\alpha \partial_\tau q_\alpha = \frac{\delta \mathcal{A}_T}{\delta q_\alpha} = \partial_t m_\alpha - \partial_{q_\alpha} H \tag{V.23b}$$

where free parameters ($\alpha = x, y, z$) ν_α set the relative timescales over which the variables m_α and q_α evolve.

Supplemented with the boundary conditions

$$m_\alpha(t = 0, \forall\tau) = m_{a,\alpha} \tag{V.24a}$$

$$m_\alpha(t = T, \forall\tau) = m_{b,\alpha} \tag{V.24b}$$

$$q_\alpha(t = 0, \forall\tau) = 0 \tag{V.24c}$$

$$q_\alpha(t = T, \forall\tau) = 0 \tag{V.24d}$$

the above equations will converge to the Hamilton-Jacobi equations (V.16) with fixed initial and final conditions, hence the instanton.

For numerical implementation, we introduce the fields

$$u_\alpha = m_\alpha + \nu_\alpha q_\alpha \tag{V.25a}$$

$$v_\alpha = m_\alpha - \nu_\alpha q_\alpha \tag{V.25b}$$

that evolve according to

$$\partial_\tau u_\alpha = \partial_\tau m_\alpha + \nu_\alpha \partial_\tau q_\alpha = \partial_t u_\alpha + \left(\nu_\alpha \partial_{m_\alpha} - \partial_{q_\alpha}\right) H \tag{V.26a}$$

$$\partial_\tau v_\alpha = \partial_\tau m_\alpha - \nu_\alpha \partial_\tau q_\alpha = -\partial_t v_\alpha + \left(\nu_\alpha \partial_{m_\alpha} + \partial_{q_\alpha}\right) H \tag{V.26b}$$

The boundary conditions imposed upon the two fields now read

$$u_\alpha(t = T, \forall\tau) = -v_\alpha(t = T, \forall\tau) + 2m_{b,\alpha} \tag{V.27a}$$

$$v_\alpha(t = 0, \forall\tau) = -u_\alpha(t = 0, \forall\tau) + 2m_{a,\alpha} \tag{V.27b}$$

To implement the above boundary conditions, we let the fields u_α and v_α propagate backward and forward in physical time t, respectively. Specifically, at each iteration step in τ, one first updates u_α while fixing v_α by propagating the final condition (V.27a) from $t = T$ to $t = 0$ using (V.25a) and then updates v_α keeping u_α via propagating the initial condition (V.27b) from $t = 0$ to $t = T$ using (V.25b). The order in which the fields v_α, u_α are updated makes no difference.

VI. SPECTRAL AND TRAJECTORY-LEVEL METASTABILITY

In this section, we discuss the connection and distinction between metastability at the spectral and trajectory levels.

A. Approximate switching rate

In the main text, we have clarified the relation between metastability at the trajectory and spectral levels by considering the following scenario. After each stochastic switching, the system resets—meaning the density operator $\hat{\rho}_t$ is effectively reset to that of the corresponding instantaneous pure state $\hat{\psi}_t = |\psi_t\rangle\langle\psi_t|$. In the time interval between successive switching events, the ensemble density operator evolves as the average over all such pure states following the last reset.

For a real λ_1, the eigenvectors $|\alpha_l^\pm\rangle$ of the metastable states $\hat{\rho}_\pm$ form an orthonormal basis for the Hilbert space. Consequently, an arbitrary quantum state $|\psi_t\rangle$ can be expressed as a linear combination, $|\psi_t\rangle = \sum_l c_l^+ |\alpha_l^+\rangle + \sum_l c_l^- |\alpha_l^-\rangle$. Since the dominant eigenstates of $\hat{\rho}_\pm$ have excitation densities centered near $n_e^\pm$, localization within a specific metastable state (subspace)—say, $\hat{\rho}_+$—requires the coefficients c_l^- of the dominant eigenvectors of the other metastable state to vanish. This condition also leads to a vanishing occupation in the corresponding state, $D[\hat{\rho}_-, \hat{\psi}_t] = 0$. Subsequent short-time stochastic dynamics then explores the subspace spanned exclusively by $|\alpha_l^+\rangle$ until a quantum jump occurs, triggering a transition between states $\hat{\rho}_+$ and $\hat{\rho}_-$.

Consistent with this picture, as shown in Fig. 4(b) of the main text, following each stochastic switching,

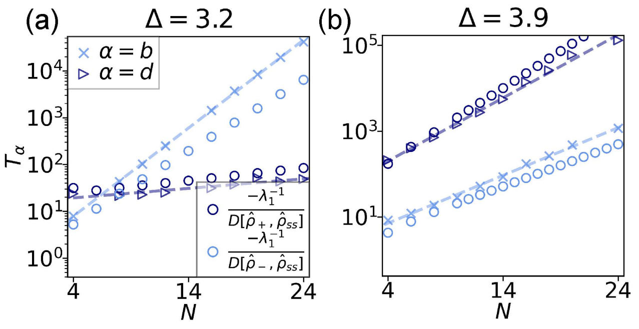

FIG. 5. Mean waiting times for the dark (T_d) and bright (T_b) states as a function of system size N for (a) $\Delta = 3.2$ and (b) $\Delta = 3.9$. Open circles of the same color indicate estimates from the gap and the state occupation $-(\lambda_1 D[\hat\rho_\pm, \hat\rho_{ss}])$. Crosses and open triangles indicate values extracted from quantum-jump Monte Carlo simulations.

there is a coordinated sharp change in both the excitation density $n_e(t)$ and the instantaneous (or initial) occupation, characterized by the normalized Hilbert-Schmidt inner product $D[\hat\rho_\pm, \hat\psi_t]$ with respect to $\hat\rho_\pm$. Although the instantaneous occupation fluctuates over time, its time average over the interval between successive jumps converges to either $D[\hat\rho_-, \hat\rho_+] = 0$ or $D[\hat\rho_+, \hat\rho_+] = D[\hat\rho_-, \hat\rho_-] = 1$. Furthermore, the dynamics of occupation between successive resets represents a fast relaxation (on timescales $\lesssim -\Re[\lambda_2]^{-1}$), followed by a latent period (on timescales $\sim -\lambda_1^{-1}$).

This demonstrates that spectral-level metastability is manifested whenever switching occurs. Specifically, the slowest relaxation manifests at the trajectory level as stochastic switching between $\hat\rho_\pm$. The ratio of the mean lifetimes T_d and T_b in the states $\hat\rho_+$ and $\hat\rho_-$ are related to the occupation probabilities $p_\pm = D[\hat\rho_\pm, \hat\rho_{ss}]$ by the relation,

$$\frac{p_+}{p_-} = \frac{T_d}{T_b} \tag{VI.1}$$

Furthermore, the switching rates are on the order of the Liouvillian gap,

$$T_d^{-1}, T_b^{-1} \propto -\lambda_1. \tag{VI.2}$$

Combining these two relations, we can intuitively express the individual switching rates as,

$$T_{b(d)}^{-1} \propto -\lambda_1 D[\hat\rho_{+(-)}, \hat\rho_{ss}] \tag{VI.3}$$

This relation simply summarizes how the spectral gap λ_1 and the stationary-state occupation ratio—both obtained from the Liouvillian spectrum—together determine the characteristic switching timescales observed in the trajectory dynamics.

As seen in Fig. 5, the lifetimes estimated from this relation are in qualitative agreement with the results from quantum-jump Monte Carlo simulations, as predicted by Eq. (VI.3). However, this relation provides only an approximate scaling of the mean switching times, and a notable quantitative discrepancy persists, especially for large N.

B. Effective barrier

We now provide a quantitative comparison of effective barriers and relaxation times across quantum and semi-classical descriptions. Fig. 6(a) shows the difference in the normalized effective energy barrier between the dark and bright states, calculated from the occupation probabilities of two respective subspaces $\hat\rho_\pm$, mean switching times, and the action increments along the instanton. They demonstrate the connection between the steady-state occupation probabilities and the lifetimes of the metastable states, which are captured in terms of the varied effective quasipotential from one state to the other. The relaxation time dictated by the switching rates is estimated through [28]

$$\tau \approx (T_b^{-1} + T_d^{-1})^{-1} \propto e^{\max\{v_b, v_d\}N} \tag{VI.4}$$

which also shows exponential size scaling. As plotted in Fig. 6(b), the exponent $\ln \tau / N$ reaches a peak around $\Phi_{bd} \approx 0$, where both states exist almost on an equal footing. It decreases as the difference between the two states increases and vanishes outside the bistable regime.

VII. RESULTS FOR RYDBERG LATTICE

We have introduced the heuristic collective jump operator $\hat L$ defined below Eq. (5) of the main text to reduce computation complexity. This approximate jump operator captures the linear dependence of the emission rate on the total Rydberg population; it does not capture the phase differences between configurations that are induced by the original local dissipation in Eq. (3) of the main

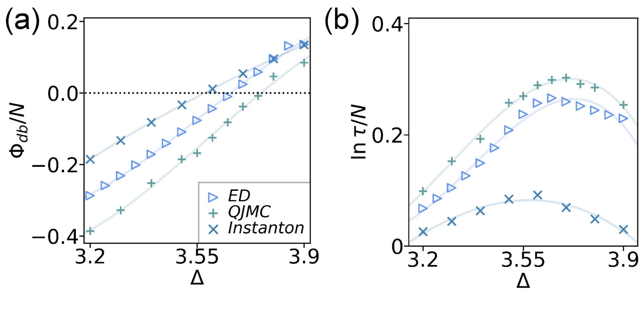

FIG. 6. (a) The difference in the normalized effective energy barrier Φ_{db} between the dark and bright states and (b) the relaxation time τ within the bistable regime estimated through the exact diagonalization (ED) of the Liouvillian, the quantum-jump Monte-Carlo (QJMC) simulations, and the instanton approach as a function of detuning Δ.

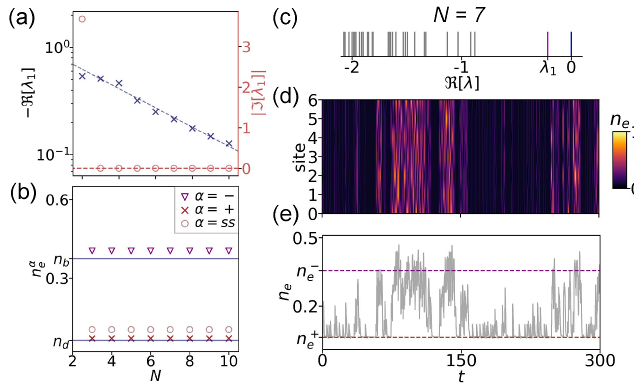

FIG. 7. Lindbladian spectrum and quantum trajectory for system with all-to-all coupling and local dissipation. (a) Real (blue crosses) and imaginary (red open circles) parts of the eigenvalue λ_1 of the first excited state $\hat{\rho}_1$ of the Lindbladian, plotted against system size N. (b) Spectral decomposition of $\hat{\rho}_1$ when λ_1 is real. The expectation values of the Rydberg populations of the steady state ($n_e^{\rm ss}$) and the quantum dark (n_e^+) and bright (n_e^-) states are shown. (c) Real parts of the eigenvalues for $N = 7$. (d) Site-resolved excitation density and (e) its spatial average from a quantum trajectory from quantum-jump Monte-Carlo simulations for $N = 7$. The excitation density $n_e^\pm$ of the two quantum metastable states $\hat{\rho}_\pm$ are marked by red and purple dashed lines. Parameters for (a)-(e) are $\Delta = 3.4$.

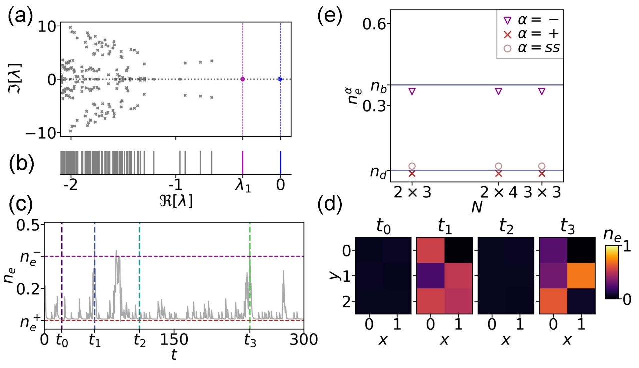

FIG. 8. Lindbladian spectrum and quantum trajectory for a 3×2 system with van der Waals coupling and local dissipation under periodic boundary conditions. (a) The first 300 eigenvalues λ_l of the Lindbladian, order by their real parts according to: $0 = \lambda_0 > \lambda_1 > \Re[\lambda_2] \geq \ldots$ The first (λ_0) and second (λ_1) eigenvalues are highlighted with blue and magenta symbols, respectively; their real parts are marked by vertical dashed lines of the same color. (b) Real parts of the eigenvalues displayed in (a), emphasizing λ_0 (blue) and λ_1 (magenta). (c) Site-resolved excitation density and (d) its spatial average from a quantum trajectory from quantum-jump Monte-Carlo simulations. The excitation density $n_e^\pm$ of the two quantum metastable states $\hat{\rho}_\pm$ are marked by red and purple dashed lines. (e) Excitation populations $n_e^\pm$ as a function of system size $N = L_x L_y$. MF dark (n_d) and bright (n_b) states are marked by blue lines. Parameters for (a)-(e) are $\Delta = 3.4$.

text. Consequently, it introduces additional dissipative terms for high-excitation states [see Sec. I].

A. All-to-all interaction

To confirm that neglecting these phase differences does not alter the fundamental physics—specifically, the exponential size scaling and the correspondence between the spectrum- and trajectory-level metastability—we performed exact diagonalization (spectral decomposition) and quantum-jump Monte-Carlo simulations using the full local dissipation of Eq. (3) with all-to-all interactions. Due to the severe computational complexity [$\mathcal{O}(4^N)$], this was limited to systems of $N = 2 \sim 10$ atoms, as presented in Fig. 7.

As shown in Fig. 7(a), the relevant eigenvalue λ_1 is complex for the smallest interacting system ($N = 2$) and becomes real for $N \geq 3$. For cases when λ_1 is real, the spectral decomposition of $\hat{\rho}_1$ reveals the quantum dark ($\hat{\rho}_+$) and bright ($\hat{\rho}_-$) states, whose Rydberg populations n_e^+ and n_e^- are located near the corresponding mean-field stable fixed points n_d and n_b, as indicated by the blue horizontal lines therein.

The emergence of bistability-induced metastability, manifested as a separation of timescales between the first two eigenmodes $\hat{\rho}_1$ and $\hat{\rho}_{\rm ss}$, is also evident in the real part of the eigenspectrum, plotted in Fig. 7(c). This spectral structure directly governs the trajectory dynamics: individual quantum trajectories exhibit stochastic switching between the corresponding metastable states. As shown in Figs. 7(d) and (e), these switching events trigger nearly synchronized excitation and de-excitation of all atoms, which is clearly visible in the site-resolved data.

B. van der Waals interaction

Last but not the least, the general connection we establish between spectrum-level metastability (a real Liouvillian gap and the quantum metastable states) and trajectory-level metastability (stochastic switching) extends beyond all-to-all interactions. Switching dynamics also occurs in systems with finite-range interactions, such as those governed by van der Waals couplings, where the quantum dark and bright states persist.

Fig. 8 presents results for a 3×2 system with van der Waals interactions and local dissipation under periodic boundary conditions. As shown in Figs. 8(a) and (b), the Liouvillian spectrum reveals a real second eigenvalue λ_1, whose corresponding eigenmatrix $\hat{\rho}_1$ is Hermitian and possesses the structure $\hat{\rho}_1 \propto \hat{\rho}_+ - \hat{\rho}_-$, clearly signaling the two metastable states $\hat{\rho}_\pm$.

Although the separation of timescales between the first two eigenmodes and the remainder is less pronounced than in the all-to-all coupling case, the same dynamical signatures of metastability are observed. As predicted by this spectral picture, an individual quantum-jump Monte-Carlo trajectory exhibits clear stochastic switch-

ing between the quantum dark and bright states, with coordinated excitation and de-excitation of atoms, as shown in Figs. 8(c) and (d).

Notably, the excitation densities $n_e^{\pm}$ of $\hat{\rho}_{\pm}$ states remain localized near the MF dark and bright states and are insensitive to system size [see Fig. 8(e)]. Compared with the all-to-all case, both the lifetime and the Rydberg density of the bright state are lower under van der Waals couplings. This reduction in lifetime and the renormalization of the excitation density likely stem from enhanced spatial fluctuations in the finite-range interaction profile.

[1] F. Minganti, A. Biella, N. Bartolo, and C. Ciuti, Spectral theory of liouvillians for dissipative phase transitions, Phys. Rev. A **98**, 042118 (2018).

[2] M. B. Plenio and P. L. Knight, The quantum-jump approach to dissipative dynamics in quantum optics, Rev. Mod. Phys. **70**, 101 (1998).

[3] C. W. Gardiner, A. S. Parkins, and P. Zoller, Wave-function quantum stochastic differential equations and quantum-jump simulation methods, Phys. Rev. A **46**, 4363 (1992).

[4] J. Dalibard, Y. Castin, and K. Mølmer, Wave-function approach to dissipative processes in quantum optics, Phys. Rev. Lett. **68**, 580 (1992).

[5] J. P. Garrahan and I. Lesanovsky, Thermodynamics of quantum jump trajectories, Phys. Rev. Lett. **104**, 160601 (2010).

[6] H. Touchette, The large deviation approach to statistical mechanics, Phys. Rep. **478**, 1 (2009).

[7] L. Causer, I. Lesanovsky, M. C. Bañuls, and J. P. Garrahan, Dynamics and large deviation transitions of the XOR-Fredrickson-Andersen kinetically constrained model, Phys. Rev. E **102**, 052132 (2020).

[8] S. Whitelam and D. Jacobson, Varied phenomenology of models displaying dynamical large-deviation singularities, Phys. Rev. E **103**, 032152 (2021).

[9] C. Ates, B. Olmos, J. P. Garrahan, and I. Lesanovsky, Dynamical phases and intermittency of the dissipative quantum Ising model, Phys. Rev. A **85**, 043620 (2012).

[10] R. H. Dicke, Coherence in spontaneous radiation processes, Phys. Rev. **93**, 99 (1954).

[11] F. Carollo and C. Pérez-Espigares, Entanglement statistics in Markovian open quantum systems: A matter of mutation and selection, Phys. Rev. E **102**, 030104 (2020).

[12] J. P. Garrahan, R. L. Jack, V. Lecomte, E. Pitard, K. van Duijvendijk, and F. van Wijland, Dynamical first-order phase transition in kinetically constrained models of glasses, Phys. Rev. Lett. **98**, 195702 (2007).

[13] K. Macieszczak, M. Guţă, I. Lesanovsky, and J. P. Garrahan, Towards a theory of metastability in open quantum dynamics, Phys. Rev. Lett. **116**, 240404 (2016).

[14] M. Marcuzzi, M. Buchhold, S. Diehl, and I. Lesanovsky, Absorbing state phase transition with competing quantum and classical fluctuations, Phys. Rev. Lett. **116**, 245701 (2016).

[15] M. Buchhold, B. Everest, M. Marcuzzi, I. Lesanovsky, and S. Diehl, Nonequilibrium effective field theory for absorbing state phase transitions in driven open quantum spin systems, Phys. Rev. B **95**, 014308 (2017).

[16] M. O. Scully and M. S. Zubairy, *Quantum Optics*, 1st ed. (Cambridge university press, 1997).

[17] H.-K. Janssen, On a Lagrangean for classical field dynamics and renormalization group calculations of dynamical critical properties, Z. Physik B **23**, 377 (1976).

[18] P. C. Martin, E. D. Siggia, and H. A. Rose, Statistical dynamics of classical systems, Phys. Rev. A **8**, 423 (1973).

[19] R. Zakine and E. Vanden-Eijnden, Minimum-action method for nonequilibrium phase transitions, Phys. Rev. X **13**, 041044 (2023).

[20] A. Kamenev, *Field theory of non-equilibrium systems*, 2nd ed. (Cambridge University Press, 2023).

[21] S. Coleman, Quantum tunneling and negative eigenvalues, Nucl. Phys. B **298**, 178 (1988).

[22] M. I. Dykman, E. Mori, J. Ross, and P. M. Hunt, Large fluctuations and optimal paths in chemical kinetics, J. Chem. Phys. **100**, 5735 (1994).

[23] V. Elgart and A. Kamenev, Rare event statistics in reaction-diffusion systems, Phys. Rev. E **70**, 041106 (2004).

[24] R. Graham and T. Tél, Nonequilibrium potential for coexisting attractors, Phys. Rev. A **33**, 1322 (1986).

[25] J. I. Park, B. J. Kim, and H. J. Park, Stochastic resonance of abundance fluctuations and mean time to extinction in an ecological community, Phys. Rev. E **104**, 024133 (2021).

[26] P. C. Bressloff and J. M. Newby, Path integrals and large deviations in stochastic hybrid systems, Phys. Rev. E **89**, 042701 (2014).

[27] A. I. Chernykh and M. G. Stepanov, Large negative velocity gradients in Burgers turbulence, Phys. Rev. E **64**, 026306 (2001).

[28] R. M. Wilson, K. W. Mahmud, A. Hu, A. V. Gorshkov, M. Hafezi, and M. Foss-Feig, Collective phases of strongly interacting cavity photons, Phys. Rev. A **94**, 033801 (2016).
